# Uncommon Bis-Amide Matrine-type Alkaloids From *Sophora alopecuroides* With Anti-inflammatory Effects

**DOI:** 10.3389/fchem.2021.740421

**Published:** 2021-09-15

**Authors:** Ding Luo, Zhenchao Tu, Wenjing Yin, Chunlin Fan, Nenghua Chen, Zhongnan Wu, Weilong Ding, Yaolan Li, Guocai Wang, Yubo Zhang

**Affiliations:** ^1^ Guangdong Province Key Laboratory of Pharmacodynamic Constituents of TCM and New Drugs Research, Institute of Traditional Chinese Medicine & Natural Products, College of Pharmacy, Jinan University, Guangzhou, China; ^2^ Department of Neurosurgery, The First Affiliated Hospital of Jinan University, Guangzhou, China; ^3^ Guangdong Clinical Translational Center for Targeted Drug, Department of Pharmacology, School of Medicine, Jinan University, Guangzhou, China

**Keywords:** matrine-type alkaloids, water-soluble alkaloids, *Sophora* alopecuroides, structure modification, structure elucidation

## Abstract

Four new alkaloids (1–4) belonging to rare examples of bis-amide matrine-type were isolated from the seeds of sophora alopecuroides. Their structures including absolute configuration were determined by extensive spectroscopic analysis, electronic circular dichroism (ECD) interpretation, and X-ray diffraction crystallography. Chemically, bis-amide matrine-type alkaloids can provide new molecular template for structural modification. Compounds 3–4 displayed obvious anti-inflammatory effects based on the inhibition of two key pro-inflammatory cytokines [tumor necrosis factor-α (TNF-α) and interleukin-6 (IL-6)] in a dose-dependent manner, with IC50 values from 35.6 to 45.8 μm.

## Introduction

*Sophora alopecuroides* L. which belongs to the family of Leguminosae, is a salt-tolerant perennial herb plant and distributed in arid desert grassland of northwest China (Wang et al., 2012; Deng et al., 2019). The seeds of *S. alopecuroides* (Chinese name: Ku-Dou-Zi) has been regarded as a well-known traditional Chinese medicine for the treatment of fever, rheumatism, bacterial infection and inflammatory diseases (Huang et al., 2016). Previous phytochemical investigations on *S. alopecuroides* revealed alkaloids as one of principal active chemical constituents. Among them, matrine-type alkaloids exhibit diverse bioactivities such as antiviral (Zou et al., 2020), anti-insect (Huang et al., 2017), anti-tumor (Li et al., 2020), and promising anti-inflammatory activities (Huang et al., 2016; Li et al., 2020).

Chemically, matrine-type alkaloids are considered as ideal lead compounds for further structure modifications because of special chemical structure, widespread biological activities, high safety threshold, as well as available commercial sources. In order to improve the activities and amplify their applicants, many matrine-type derivatives have been synthesized and reported in the recent years (Huang et al., 2017; Cai et al., 2018; Cheng et al., 2018; Lv et al., 2018; Cheng et al., 2020; Xu et al., 2020). Interestingly, we have noticed that almost all structural modifications were concentrated in the variations of D ring, such as introducing substituents to C-13 and C-14 sites, opening D ring, fusing D ring and further molecular simplification. However, the amide bond located at D ring is a critical part that can responsible for many biological activities according to the molecular docking analysis (Peng et al., 2020). It is necessary to search more matrine-type template for the development of structural modification strategy.

**FIGURE 1:**
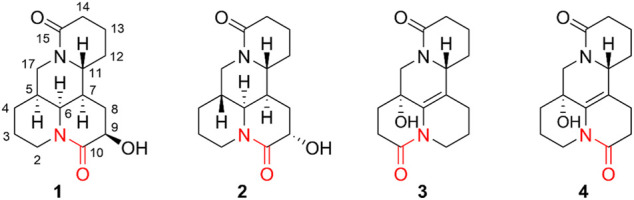
Chemical structures of compounds 1–4.

As a part of continuous systematic search for structurally unique and biologically meaningful natural products from the *sophora* species (Fan et al., 2019; Luo et al., 2021; Zhang et al., 2018a; Zhang et al., 2018b; Zhang et al., 2016; Zhang et al., 2017), four new matrine-type alkaloids (1–4) were obtained and identified (Figure 1). It is worth mentioning that compounds 1–4 are uncommon examples of bis-amide matrine with the second amide group at C-2 or C-10. To data, hundreds of matrine-type alkaloids have been reported but only few cases such as 2-oxymatrine, 10-oxy-5,6-dehydromatrine, and 10-oxysophoridine that possessed the bis-amide bond at C-2 or C-10 (Zhang et al., 2016; Zhang et al., 2018a). Additionally, all isolates were evaluated their anti-inflammatory activities *in vitro* based on production of two key pro-inflammatory cytokines (TNF-*α* and IL-6) in lipopolysaccharides (LPS)-stimulated RAW264.7 cells. Herein, the isolation, strucutre elucidation, and anti-inflammatory activites of those isolates are discussed.

## Materials and Methods

### General Experimental Procedures

The UV spectra, optical rotations, and IR spectra were determined on a JASCO V-550 UV/VIS spectrophotometer in MeOH solutions, an Autopol JASCO P-1020 polarimeter with concentrations unit reported in g/100 ml, a JASCO FT/IR-480 plus FT-IR spectrometer with KBr disks and peaks reported in cm^−1^ (JASCO corporation, Tokyo, Japan), respectively. ECD spectrum were carried out on a JASCO J-810 spectrometer. NMR experiments including 1D and 2D spectra were run on a Bruker Avance 600 NMR (600 MHz for ^1^H/150 MHz for ^13^C) spectrometer using standard Bruker pulse sequences (Bruker-Biospin, United States). HRESIMS data were measured from an Agilent 6210 LC/MSD Q-TOF mass instrument (Agilent Technologies, CA, United States). Analysis and preparation of crude samples were performed on HPLC using Shimadzu 6AD series with a PDA detector (Shimadzu corporation, Tokyo, Janpan). Al_2_O_3_ thin layer chromatography (TLC) analysis was done on aluminium oxide 60 F 254 basic plates (Merck, China). Column chromatography (CC) was undertaken with D-101 macroporous resin (Diaion, Shanghai, China), simon alumina N (size 200–300 mesh, Aldrich, China), Sephadex LH-20 (size 25–100 mm, Fluka, Buchs, Switzerland), CHP20P MCI gel (size 75–150 μm, Sigma-Aldrich company Ltd. China), and ODS silica gel (size 50 mm; YMC, Tokyo, Japan).

### Plant Material

The dry seeds of *S. alopecuroides* L. were collected from the area (GPS coordinates, 37°98′−38°22′ N, 106°20′−106°66′ E) of Wuzhong City, Ningxia Hui Autonomous Region, People’s Republic of China on August 2014. A voucher specimen (accession no. SA-2014–08–28) was authenticated by Prof. Guang-Xiong Zhou (Jinan University) and available for inspection at the Institute of Traditional Chinese Medicine and Natural Products, Jinan University, Guangzhou, P. R. China.

### Extraction and Isolation

The dry seeds of *S. alopecuroides* (∼30 kg) were powdered and percolated thrice with 95% EtOH and the pooled extracts were concentrated to give the total extract (∼1.9 kg). The crude residue was suspended with 0.1 M aqueous HCl until acidified to pH 2–3. After removal of the nonalkaloid components with CHCl_3_, the remaining acidic solvent was subsequently adjusted to pH 9–10 basified with saturated NH_3_ solution in water and partitioned with CHCl_3_ five times to yield total alkaloids (0.8 kg, extraction coefficient: 2.67%). The CHCl_3_ layer alkaloid was fractionated via D−101 macroporous resin CC eluting with EtOH−H_2_O (from 10:90 to 95:5, *v*:*v*) to yield five major fractions (Fr.1−5), based on the ratio of ethanol and water. Fr. 1 was dissolved in deionized water, and extracted twice with ethyl acetate (EtOAc) to remove the fat-soluble part. Subsequently, Fr. 1A (50.1 g) was subjected to Al_2_O_3_-based chromatography column (200–300 mesh) eluted with CH_2_Cl_2_ containing increasing amount of MeOH (100:0 to 0:100, *v/v*) added 1% Et_2_NH to produce seven portions (Fr.1Aa–Fr.1Ag), based on Al_2_O_3_-based TLC analysis. Fr.1Af (18.0 g) was separated on a RP-MCI column chromatography with a gradient of CH_3_OH/H_2_O/Et_2_NH (5:95:0.01 to 60:40:0.01) to generated five fractions (Fr.1Af.1−5). Fr.1Af.1 was applied to a Sephadex LH−20 column (CH_3_OH/H_2_O, 2:1, *v/v*) to obtain three subfractions (Fr.1Af.1.1–Fr.1Af.1.3). Fr.1Af.1.2 was purified by ODS CC (CH_3_OH/H_2_O, 5:95→40:60, *v/v*) and further separated by preparative HPLC with CH_3_CN/H_2_O/Et_2_NH (15:85:0.01, *v/v/v*) to give compounds 1 (19.4 mg, *t*_R_ = 21.4 min), 2 (11.1 mg, *t*_R_ = 23.8 min), 3 (23.2 mg, *t*_R_ = 17.8 min), 4 (24.9 mg, *t*_R_ = 16.9 min).

(+)-10-Oxy-9*β*-hydroxymatrine (1): colorless crystals in MeOH; mp 147–148°C [*α*]_25_^D^ +84.0 (*c* 0.01, CH_3_OH); UV (CH_3_OH) *λ*_max_ (log *ε*) 205 (3.52) nm; IR (KBr) *ν*_max_ 3,252, 2,927, 2,867, 1,627, 1,597, 1,408, 1,058 cm^−1^; ^1^H and ^13^C NMR data, see Table 1; HRESIMS *m/z* 279.1698 [M + H]^+^ (calcd for C_15_H_23_N_2_O_3_, 279.1703).

**TABLE 1:** ^1^H (600 MHz) and^13^C NMR (150 MHz) data of compounds one to four in CDCl_3_ (*δ* in ppm, *J* in Hz)^a^.

No	1		2		3		4	
	*δ*_C_	*δ*_H_	*δ*_C_	*δ*_H_	*δ*_C_	*δ*_H_	*δ*_C_	*δ*_H_
2	42.3	a 4.55 dd (11.6, 2.5); b 2.73	44.1	a 4.69 dd (13.2, 4.4); b 2.59 td (13.2, 2.9)	167.8		39.5	a 3.76; b 3.76
3	19.7	a 1.66; b 1.66	25.0	a 1.79; b 1.53	27.8	a 2.92 m; b 2.54	20.4	a 1.93; b 1.62
4	26.5	a 1.79; b 1.67	29.8	a 1.94; b 1.29	29.7	a 1.96 m; b 1.76	32.0	a 1.88; b 1.84
5	34.4	1.94	35.8	1.83	65.1		68.1	
6	57.0	3.54 days (3.4)	59.6	3.27 dd (11.0, 9.0)	133.2		134.9	
7	40.7	1.83	36.1	2.34	113.7		117.2	
8	29.3	a 2.18; b 1.95	28.4	a 2.13; b 1.87	24.1	a 2.20 m; b 1.87 dd (17.0, 4.8)	21.3	a 2.39; b 2.09
9	66.8	4.18 days (5.9)	64.3	4.01 dd (10.4, 4.2)	21.3	a 2.00; b 1.63	32.4	a 2.55; b 2.48
10	172.8		170.5		39.7	a 4.71 days (13.2); b 2.78 t (13.2)	168.9	
11	54.0	3.77 m	53.3	3.67 m	58.6	3.92 dd (11.3, 3.8)	56.8	4.12 (11.3, 4.1)
12	27.4	2.41 m; 1.45	27.7	2.21; 1.43	28.3	a 2.33 days (13.5); b 1.49 m	26.8	a 2.22; b 1.35
13	19.0	a 1.81; b 1.66	19.1	a 1.93; b 1.67	20.0	a 1.97; b 1.75 m	19.4	a 1.88; b 1.73
14	32.9	a 2.44; b 2.26	32.3	a 2.46 m; b 2.34	32.2	a 2.55 m; b 2.43	31.2	a 2.38; b 2.32
17	169.7	*α* 4.46 dd (13.2, 4.1); *β* 2.71 t (13.2)	170.2	α 3.65 m; β 3.14 t (13.2)	170.7	*α* 4.81 days (13.2); *β* 2.67 days (13.2)	170.0	*α* 4.63 days (13.2); *β* 2.90 days (13.2)

a Overlapped signals were reported without designating multiplicity.

(−)-10-Oxy-9*α*-hydroxysophoridine (2): pale yellow oil in MeOH [*α*]_25_^D^ −29.2 (*c* 0.01, CH_3_OH); UV (CH_3_OH) *λ*_max_ (log *ε*) 206 (3.33) nm; ECD (CH_3_OH) *λ*_max_ (Δ*ε*) 228 (+1.9) nm; IR (KBr) *ν*_max_ 3,412, 3,256, 2,932, 2,867, 1,625, 1,603, 1,409, and 1,186 cm^−1^; ^1^H and ^13^C NMR data, see Table 1; HRESIMS *m/z* 279.1702 [M + H]^+^ (calcd for C_15_H_23_N_2_O_2_, 279.1703).

(−)-2-Oxy-5-hydroxy-6,7-dehydromatrine (3): colorless block crystals in MeOH; mp 135–136°C [*α*]_25_^D^ −8.9 (*c* 0.01, CH_3_OH); UV (CH_3_OH) *λ*_max_ (log *ε*) 201 (4.08), 242 (3.11) nm; IR (KBr) *ν*_max_ 3,408, 2,925, 2,765, 1,626, 1,459, and 1,098 cm^−1^; ^1^H and ^13^C NMR data, see Table 1; HRESIMS *m/z* 277.1541 [M + H]^+^ (calcd for C_15_H_21_N_2_O_3_, 277.1547).

(−)-10-Oxy-5-hydroxy-6,7-dehydromatrine (4): pale yellow oil in MeOH [*α*]_25_^D^ −12.2 (*c* 0.01, CH_3_OH); UV (CH_3_OH) *λ*_max_ (log *ε*) 206 (3.25) nm; ECD (CH_3_OH) *λ*_max_ (Δ*ε*) 209 (−1.9), 228 (+2.5), 257 (−4.8) nm; IR (KBr) *ν*_max_ 3,408, 2,933, 2,803, 1,623, 1,441, 1,193 cm^−1^; ^1^H and ^13^C NMR data, see Table 1; HRESIMS *m/z* 277.1546 [M + H]^+^ (calcd for C_15_H_21_N_2_O_3_, 277.1547).

### Single X-Ray Diffraction Data Analysis

The single-crystal X-ray diffraction data of one and three were acquired on an Agilent diffractometer with Cu K*α* radiation. The structures were solved by the SHELXT structure solution program and refined with full-matrix least-squares minimization on *F* ^2^ using SHELXL via OLEX2 software package. Crystallographic data for one and three have been deposited in the Cambridge Crystallographic Data Centre (free of charge at https://www.ccdc.cam.ac.uk/) under deposition numbers CCDC are 2055031 and 2095514, respectively.

Crystal Data for (−)-10-oxy-9*β*-hydroxymatrine (1): C_15_H_24_N_2_O_4_ (*M* = 296.36 g/mol): orthorhombic, space group *P2*_*1*_ *2*_*1*_ *2*_*1*_ (no. 19), *a* = 8.4437 (2) Å, *b* = 10.5310 (3) Å, *c* = 33.5834 (7) Å, *V* = 2,986.26 (13) Å^3^, *Z* = 8, *T* = 149.99 (10) K, *μ*(Cu K*α*) = 0.784 mm^−1^, *Dcalc* = 1.318 g/cm^3^, 13,927 reflections measured (5.262° ≤ 2*Θ* ≤ 147.692°), 5,895 unique (*R*_int_ = 0.0312, *R*_*s*igma_ = 0.0387) which were used in all calculations. The final *R*_1_ was 0.0385 (*I* > 2*σ*(*I*)) and *wR*_2_ was 0.0912 (all data). Flack parameter −0.09 (10). Hooft parameter: −0.06 (10). CCDC number is 2055031.

Crystal Data for (−)-2-oxy-5-hydroxy-6,7-dehydromatrine (3): C_15_H_22_N_2_O_4_ (*M* = 294.34 g/mol): orthorhombic, space group P2_1_2_1_2_1_ (no. 19), *a* = 7.41520 (10) Å, *b* = 10.77360 (10) Å, *c* = 17.6626 (2) Å, *V* = 1,411.04 (3) Å^3^, *Z* = 4, *T* = 150.00 (10) K, μ(CuKα) = 0.829 mm^−1^, *Dcalc* = 1.386 g/cm^3^, 9,266 reflections measured (9.616° ≤ 2*Θ* ≤ 147.622°), 2,781 unique (*R*_int_ = 0.0197, R_sigma_ = 0.0155) which were used in all calculations. The final *R*_1_ was 0.0285 (I > 2σ(I)) and *wR*_2_ was 0.0714 (all data). Flack parameter −0.01 (6). Hooft parameter: −0.05 (5). CCDC number is 2095514.

### Electronic Circular Dichroism Calculations

Conformational analysis was initially performed using sybyl-X-2.1.1 program. Conformers occurring within a 10 kcal/mol energy window from the global minimum were chosen for geometry optimization and energy calculation using DFT with the B3LYP functional and the 6–311++G (d,p) basis set with the Gaussian09 program. Calculated results were completed using TD-DFT with the CPCM model in MeOH based on the optimized geometries. Finally, the Boltzmann-averaged ECD spectra of the two compounds were obtained and visualized with *SpecDis* 1.61 and drawn using the Origin Pro nine program. The half bandwidth (*σ*) and UV correction values applied in *SpecDis* for the final calculated ECD spectra are 0.30 eV and −5 nm, 0.30 eV and −6 nm for compounds 2 and 4, respectively, which are within the reasonable range. Their optimized conformation geometries, thermodynamic parameters, and populations of all conformations (2 and 4) are provided in supporting information.

### *In vitro* Cytotoxicity Assay

The cytotoxicity of those isolated alkaloids on RAW 264.7 cells were evaluated by *3-*(*4,5-*dimethylthiazol-2-yl)-2,5-diphenyltetrazolium Bromide (MTT) assay. Various concentrations of test alkaloids were used to treat the cells by the methods that published previously (He et al., 2019; Zhang et al., 2021).

### *In vitro* Anti-inflammatory Assay

The anti-inflammatory activities of 1–4 on LPS-stimulated TNF-*α* and IL-6 expression in RAW 264.7 cells were assessed using enzyme-linked immunosorbent assay (ELISA) as published previously (He et al., 2019). Dexamethasone and the medium without LPS were selected as the positive or negative control group.

## Results and Discussion

Compound 1 was isolated as colorless block crystals from CH_3_OH, mp 147–148°C [*α*]_25_^D^ +84.0 (*c* 0.01, CH_3_OH). The molecular formula was deduced to be C_15_H_22_N_2_O_3_ on the basis of the HRESIMS protonated molecular ion peak [M + H]^₊^ at *m/z* 278.1630, suggesting six indices of hydrogen deficiency. The UV spectrum showed maximum absorption at 206 nm was typical for non-conjugated amide of matrine-type backbone. The IR spectrum displayed a hydroxy group (3,202 cm^−1^) and two lactam functionalities (1,627 and 1,597 cm^−1^). The ^1^H NMR spectrum (Table 1) exhibited signals for [*δ*_H_ 4.70 dd (*J* = 13.2, 4.6 Hz), 2.69 m] and [*δ*_H_ 4.53 days (*J* = 13.0 Hz), 2.66 m], ascribed to two sets diagnostic methylene protons adjacent to N-atom of amide bond. The ^13^C NMR spectrum (Table 1) revealed 15 carbon resonances categorized by DEPT experiments as two carbonyls (*δ*_C_ 172.4 and 170.3), five methines (*δ*_C_ 74.5, 54.6, 54.3, 42.0, and 31.5), and eight methylenes (*δ*_C_ 46.7, 32.4, 32.1, 28.4, 27.9, 25.3, 20.0, 18.9). The above spectroscopic data as well as biogenetic considerations indicated that compound 1 was a derivative of matrine (Zhang et al., 2018b).

**FIGURE 2:**
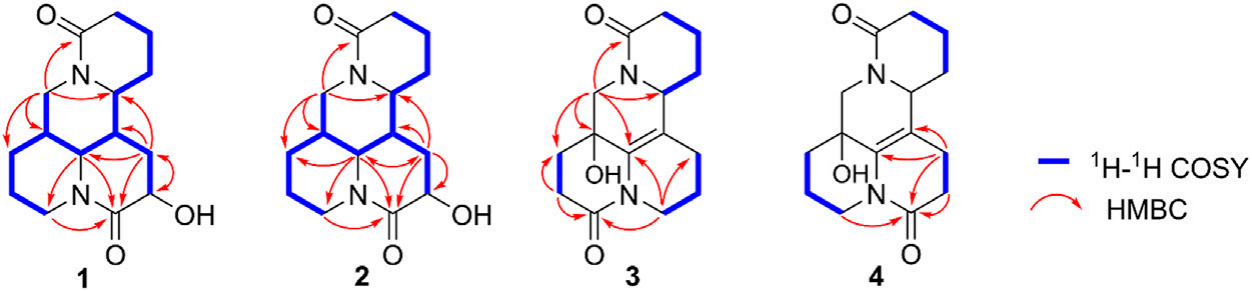
Key ^1^H−^1^H COSY and HMBC correlations of 1–4.

**FIGURE 3:**
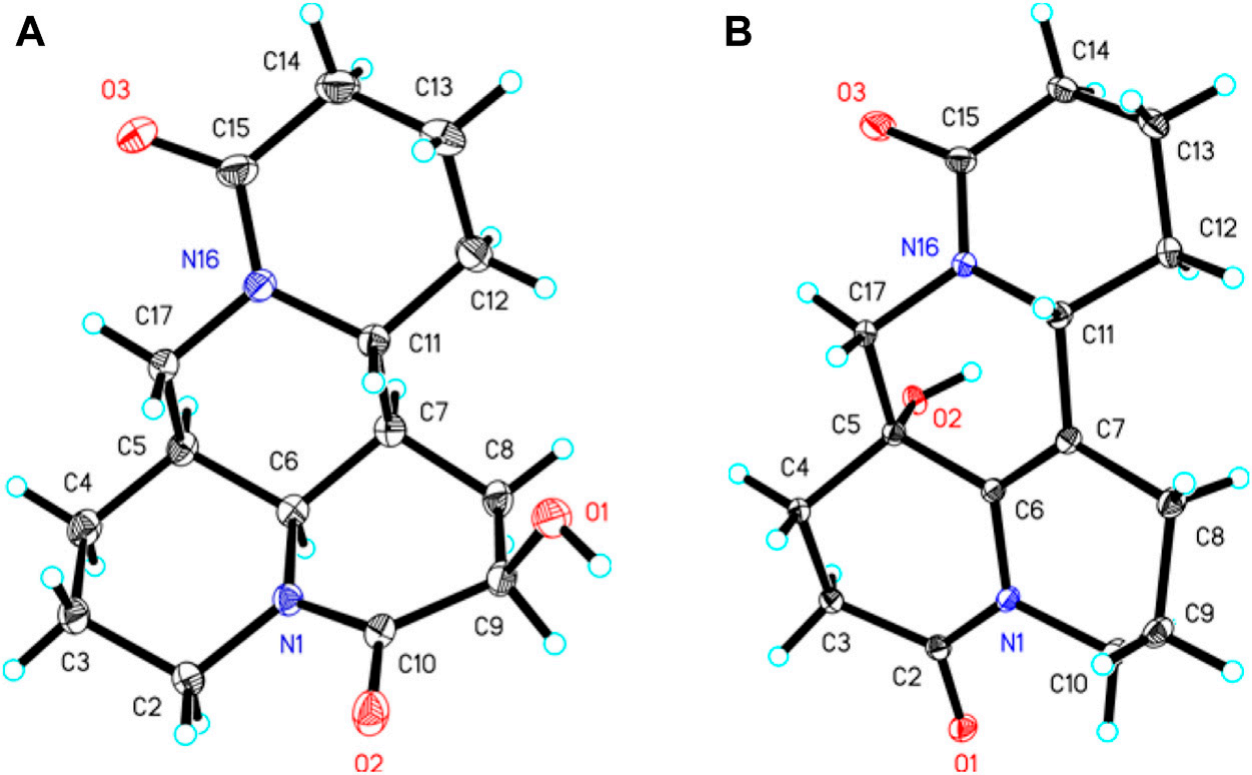
X-ray ORTEP drawings of 1 **(A)** and 3 **(B)**.

The planar structure and relative configuration of one was ascertained by comprehensive analysis of 2D NMR experiment (including ^1^H–^1^H COSY, HSQC, HMBC, and NOESY spectra). The characteristic HMBC correlations from H_2_-17 to C-4/C-5/C-6/C-11/C-17, from H-6 to C-2/C-5/C-7/C-10/C-11, from H_2_-8 to C-6/C-7/C-9/C-10/C-11, and from H-9 to C-7/C-8/C-10, together with ^1^H−^1^H COSY correlations of H-6/H-7/H-8/H-9 in B ring (Figure 2), implied one more amide bond occurring at C-10 and a hydroxy group locating on C-9 of matrine-based skeleton. Furthermore, the coupling constant between H-9 and H_2_-8 (^3^ *J* = 5.6 Hz) suggested H-9 occupied the equatorial position (*α*) in six-member ring, supported by the absence of H-9/H-11 cross-peak signal in the NOESY spectrum. Finally, the absolute configuration of (5*S*,6*S*,7*,9*,11*R*)-1 was unequivocally established by a single crystal X-ray diffraction (Figure 3) using Cu K*α* radiation [Flack parameter: 0.09 (13)]. Thus, the structure of one was deduced as shown in Figure 1, and named as (+)-10-oxy-9*β*-hydroxymatrine.

**FIGURE 4:**
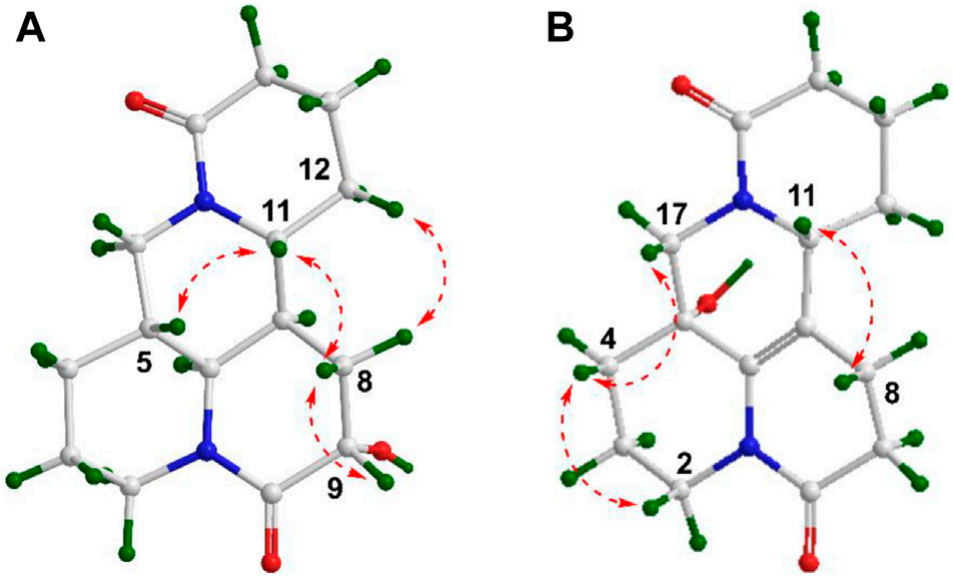
Key NOESY correlations of 2 **(A)** and 4 **(B)**.

**FIGURE 5:**
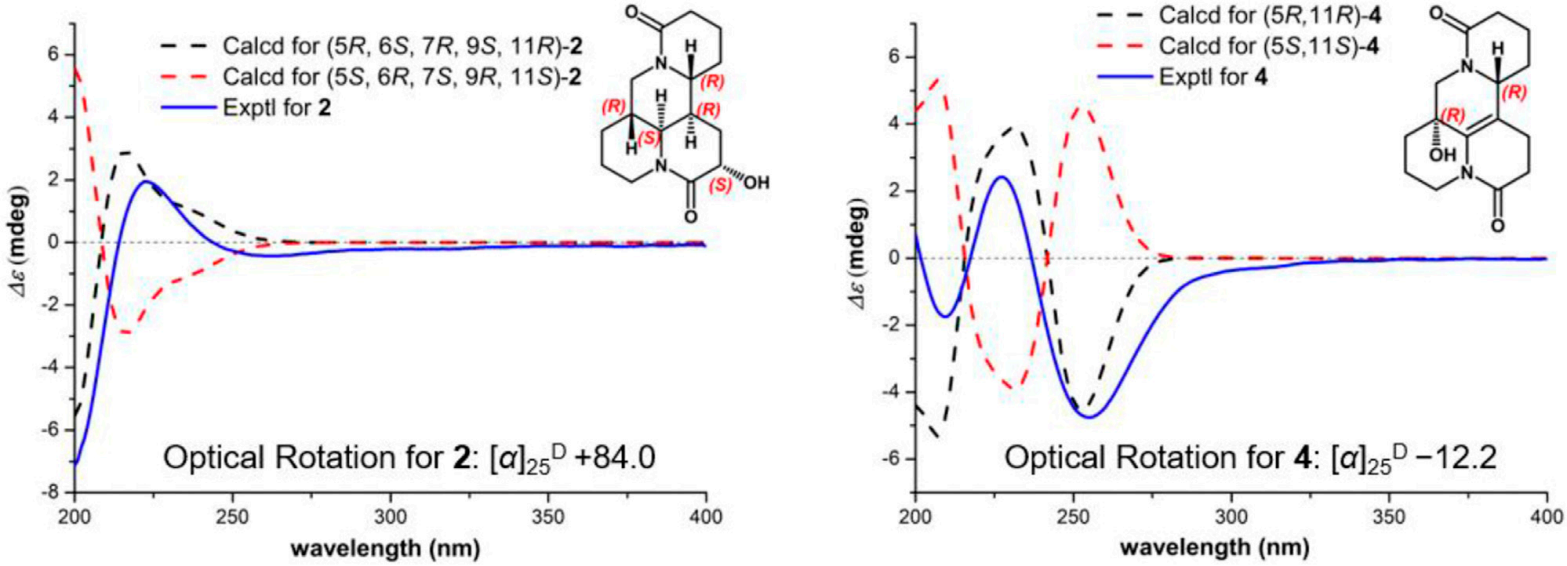
Experimental ECD and calculated spectra of 2 and 4 at the B3LYP/6–31+G (d) level [2: σ = 0.30 eV, UV shift = −5 nm; 4: σ = 0.30 eV, UV shift = −6 nm].

Compound 2 [*α*]_25_^D^ −29.2 (*c* 0.01, CH_3_OH), possessed a molecular formula of C_15_H_22_N_2_O_3_ (calcd for C_15_H_23_N_2_O_3_, 279.1703) via the HRESIMS ion peak at *m/z* 279.1704 [M + H]^+^ and ^13^C NMR data. Its spectroscopic values showed that two is structurally similar to sophoridine (Zhang et al., 2018a), while the obviously differences were that an additional amide carbon (*δ*_C_ 170.5) and a more *O*-bearing methine [*δ*_C_ 64.3, *δ*_H_ 4.01 dd (*J* = 10.4, 4.2 Hz)] are observed. The key HMBC cross-peaks of H_2_-8 to C-6/C-7/C-9/C-10/C-11 and H-6 to C-2/C-4/C-8/C-10, together with sequential COSY correlations of H-6/H-7/H_2_-8/H-9 in B ring (Figure 2) lead to the full structural assignment of 2, as shown in Figure 1. The configuration of OH-9 was deduced as equatorial orientation (*α*), which was determined by the large coupling constant of H-8α/H-9 (*J*_H-9/H-8*α*_ = 10.4 Hz) and NOE cross-peaks (Figure 4) of H-9 (*δ*_H_ 4.01)/H-8a (*δ*_H_ 2.13)/H-11 (*δ*_H_ 3.67). Consequently, the absolute structure of two was corroborated by comparing the experiment and calculated CD curve (Figure 5). Compound 2 was thereby deduced and named as (−)-10-oxy-9*α*-hydroxysophridine.

Compound 3, colorless block crystals, showed an [M + H]^+^ ion peak at *m/z* 277.1541 (calcd for C_15_H_21_N_2_O_3_, 401.1571) in the HRESIMS spectrum, consistent with the molecular formula of C_15_H_20_N_2_O_3_. The ^1^H and ^13^C NMR data of 3 (Table 1) were similar to those of 5-hydroxy-6,7-dehydro-matrine (Zhang et al., 2018b). The most notable difference was that the methylene at C-10 was replaced by another lactam group (*δ*_C_ 172.8), which was verified by the key ^1^H–^1^H COSY correlation of H-2/H-3, and HMBC cross-peaks from H_2_-3 to C-2/C-4, from H_2_-10 to C-2/C-6/C-8, and from H_2_-17 to C-4/C-5/C-6/C-11/C-17 (Figure 2). Moreover, the planar structure and absolute configuration of three were determined by X-ray crystallography (Figure 3) with an excellent Flack parameter [−0.01 (6)]. Hence, the complete structure of three was established and named as (−)-2-oxy-5-hydroxy-6,7-dehydromatrine.

Compound 4 [*α*]_25_^D^ −12.2 (*c* 0.01, CH_3_OH), was obtained simultaneously with three in the same preparation liquid phase condition. Comprehensive analysis of its spectroscopic data indicated that four possessed the same molecular formula and almost identical 1D NMR resonances (Table 1) as that of 3, except for the second amide group exchanged from A ring to B ring. In the 2D NMR spectra of 4, the ^1^H–^1^H COSY correlations of H_2_-2/H_2_-3/H_2_-4 and H_2_-8/H_2_-9, as well as HMBCs from H_2_-2 to C-10, and from H_2_-8 to C-6/C-7/C-9 suggested the second amide group is located on B ring (Figure 2). Additionally, good consistency between the experimental CD curve and calculated ECD curve (Figure 5) allowed the assignment of (5*R*,11*R*) absolute configuration. Thus, compound 4 was elucidated and named as (−)-10-oxy-5-hydroxy-6,7-dehydromatrine.

At present, the structural modifications of matrine-type alkaloids have mainly focused on the variations of D ring due to the amide group at C-15 is an active reaction site. (Cai et al., 2018; Cai et al., 2020). Compounds 1–4 possessing rare bis-amide matrine-type motif could provide new molecular template and ideas for synthetic chemists. For example, new derivatives could be designed by the introduction of a protecting group on the D ring and subsequent structural modification via the second amide bond at C-2 or C-10 position on the A or B ring. So that the key part of D ring responsible for biological activity can be retained, and some unexpected good results may emerge.

IL-6 and TNF-α are essential mediators in inflammation processes. Owing to the extracts of *S. alopecuroides* possessing remarkable clinical effects on various kinds of inflammation, all isolated alkaloids (1–4) were evaluated for their LPS-stimulated TNF-*α* and IL-6 production in RAW 264.7 cells using ELISA. Firstly, a MTT cytotoxicity assay was used to examine the cell viability of murine RAW 264.7 cells. The results displayed that all alkaloids at the concentration ranges from 3.125 to 50 μM were non-toxic on RAW 264.7 cells after 24 h treatment. Therefore, the tested alkaloids were applied to those concentration range to perform the next experiment. The production of IL-6 and TNF-α in the culture medium of the LPS-treated group both increased significantly (*p* < 0.001) in comparison with the control group after 24 h. However, co-incubation with compounds 3–4 suppressed the secretion of IL-6 and TNF-α in a dose-dependent manner, suggesting that compounds 3–4 are inhibitor of the initial phase of the inflammatory cascade initiated by LPS stimulation. As shown in Table 2, compounds 3–4 can inhibit the expression of those two pro-inflammatory mediator secretions (TNF-α and IL-6) with IC_50_ values from 35.6 to 45.8 μm, while compounds 1–2 were inactive. In light of the structures and the anti-inflammatory activity, the existence of unsaturated double bond of Δ^6(7)^ may favor this inhibitory effect.

**TABLE 2:** Inhibitory activity of compounds 1–4 against TNF-*α* and IL-6 production in RAW 264.7 cells.

Group^a^	IC_50_ (*μ*M)	
	TNF-*α*	IL-6
**1**	>50	>50
**2**	>50	>50
**3**	35.6 ± 0.9	41.2 ± 1.3
**4**	45.8 ± 1.9	>50 ± 0.6
Dex^b^	8.8 ± 1.3	7.2 ± 0.5

a Results were expressed as means ± SD (*n* = 3).

b Dex (dexamethasone) was select as positive control.

## Conclusions

The systematic investigation of the seeds of *S. alopecuroides* led to the isolation of four uncommon bis-amide matrine-type alkaloids. Their special chemical structure can provide a new perspective for developing novel modificatory strategies based on A or B ring. The biological assay revealed two new compounds displayed obvious anti-inflammatory activity. This study not only enriched the structural diversity of matrine-type alkaloids, but also provided an attractive molecular candidate for pharmaceutical chemists.

## Data Availability

The datasets presented in this study can be found in online repositories. The names of the repository/repositories and accession number(s) can be found in the article/Supplementary Material.

## Supplementary Material

The Supplementary Material for this article including original UV, CD, IR, HRESIMS, 1D/2D NMR spectra can be found online at: https://www.frontiersin.org/articles/10.3389/fchem.2021.740421/full#supplementary-material

## References

[B1] Cai X.-H. Guo H. Xie B. (2018). Structural Modifications of Matrine-Type Alkaloids. Mini. Rev. Med. Chem. 18, 730–744. 10.2174/1389557516666161104150334 27823557

[B2] Cai X. H. Zhang H. Y. Xie B. (2020). Matrine-Family Alkaloids: Versatile Precursors for Bioactive Modifications. Min. Rev. Med. Chem. 16, 431–453. 10.2174/1573406415666190507121744 31378199

[B3] Cheng X. He H. Wang W. X. Dong F. Zhang H. Ye J. (2020). Semi-Synthesis and Characterization of Some New Matrine Derivatives as Insecticidal Agents. Pest Manag. Sci. 76, 2711–2719. 10.1002/ps.5817 32166856

[B4] Cheng X. Ye J. He H. Liu Z. Xu C. Wu B. (2018). Synthesis, Characterization and *In Vitro* Biological Evaluation of Two Matrine Derivatives. Sci. Rep. 8, 15686. 10.1038/s41598-018-33908-8 30356148 PMC6200782

[B5] Deng X. X. Wang R. Z. Wu X. L. Gao Q. X. Han L. Gao X. J. (2019). *Sophora Alopecuroides* L.: An Ethnopharmacological, Phytochemical, and Pharmacological Review. J. Ethnopharmacol. 248, 112172. 10.1016/j.jep.2019.112172 31442619

[B6] Fan C. L. Zhang Y. B. Chen Y. Xie P. Wang G. C. Tian H. Y. (2019). Alopecuroides A–E, Matrine-Type Alkaloid Dimers from the Aerial Parts of *Sophora Alopecuroides* . J. Nat. Prod. 82, 3227–3232. 10.1021/acs.jnatprod.8b01081 31747283

[B7] He L. J. Liu J. S. Luo D. Zheng Y. R. Zhang Y. B. Wang G. C. (2019). Quinolizidine Alkaloids from *Sophora Tonkinensis* and Their Anti-Inflammatory Activities. Fitoterapia 139, 104391. 10.1016/j.fitote.2019.104391 31682871

[B8] Huang J. L. Lv M. Xu H. (2017). Semisynthesis of Some Matrine Ether Derivatives as Insecticidal Agents. Rsc Adv. 7, 15997–16004. 10.1039/c7ra00954b

[B9] Huang Y. X. Wang G. Zhu J. S. Zhang R. Zhang J. (2016). Traditional Uses, Phytochemistry, and Pharmacological Properties of *Sophora Alopecuroides L* . Eur. J. Inflamm. 14, 128–132. 10.1177/1721727x16642779

[B10] Li Y. Wang G. Liu J. Ouyang L. (2020). Quinolizidine Alkaloids Derivatives from *Sophora Alopecuroides* Linn: Bioactivities, Structure-Activity Relationships and Preliminary Molecular Mechanisms. Eur. J. Med. Chem. 188, 111972. 10.1016/j.ejmech.2019.111972 31884408

[B11] Luo D. Wu Z. N. Zhang J. H. Lin Q. Chen N. H. Chen S. (2021). Sophaloseedlines A−G: Diverse Matrine-Based Alkaloids from *Sophora Alopecuroides* with Potential Anti-Hepatitis B Virus Activities. Chin. J. Chem. 39, 2555–2562. 10.1002/cjoc.202100279

[B12] Lv M. Liu G. C. Jia M. H. Xu H. (2018). Synthesis of Matrinic Amide Derivatives Containing 1,3,4-thiadiazole Scaffold as Insecticidal/acaricidal Agents. Bioorg. Chem. 81, 88–92. 10.1016/j.bioorg.2018.07.034 30118989

[B13] Peng W. Xu Y. Han D. Feng F. Wang Z. Gu C. (2020). Potential Mechanism Underlying the Effect of Matrine on COVID-19 Patients Revealed through Network Pharmacological Approaches and Molecular Docking Analysis. Arch. Physiol. Biochem. 1–8. 10.1080/13813455.2020.1817944 PMC7544918 32915649

[B14] Wang H. Q. Guo S. Qian D. W. Qian Y. F. Duan J. A. (2012). Comparative Analysis of Quinolizidine Alkaloids from Different Parts of *Sophora Alopecuroides* Seeds by UPLC–MS/MS. J. Pharmaceut. Biomed. 67, 16–21. 10.1016/j.jpba.2012.04.024 22613581

[B15] Xu J. Sun Z. Hao M. Lv M. Xu H. (2020). Evaluation of Biological Activities, and Exploration on Mechanism of Action of Matrine–Cholesterol Derivatives. Bioorg. Chem. 94, 103439. 10.1016/j.bioorg.2019.103439 31776033

[B16] Zhang B. Y. Liu D. Ji W. Y. Otsuki K. H. Higai K. J. Zhao F. (2021). Sacraoxides A–G, Bioactive Cembranoids from Gum Resin of *Boswellia Sacra* . Front. Chem. 9, 649287. 10.3389/fchem.2021.649287 33869144 PMC8044883

[B17] Zhang Y. B. Luo D. Yang L. Cheng W. He L. J. Kuang G. K. (2018a). Matrine-Type Alkaloids from the Roots of *Sophora Flavescens* and Their Antiviral Activities against the Hepatitis B Virus. J. Nat. Prod. 81, 2259–2265. 10.1021/acs.jnatprod.8b00576 30298740

[B18] Zhang Y. B. Yang L. Luo D. Chen N. H. Wu Z. N. Ye W. C. (2018b). Sophalines E-I, Five Quinolizidine-Based Alkaloids with Antiviral Activities against the Hepatitis B Virus from the Seeds of *Sophora Alopecuroides* . Org. Lett. 20, 5942–5946. 10.1021/acs.orglett.8b02637 30204454

[B19] Zhang Y. B. Zhan L. Q. Li G. Q. Wang F. Wang Y. Li Y. L. (2016). Dimeric Matrine-Type Alkaloids from the Roots of *Sophora Flavescens* and Their Anti-Hepatitis B Virus Activities. J. Org. Chem. 81, 6273–6280. 10.1021/acs.joc.6b00804 27352066

[B20] Zhang Y. B. Zhang X. L. Chen N. H. Wu Z. N. Ye W. C. Li Y. L. (2017). Four Matrine-Based Alkaloids with Antiviral Activities against HBV from the Seeds of *Sophora Alopecuroides* . Org. Lett. 19, 424–427. 10.1021/acs.orglett.6b03685 28067050

[B21] Zou J. B. Zhao L. H. Yi P. An Q. He L. X. Li Y. N. (2020). Quinolizidine Alkaloids with Antiviral and Insecticidal Activities from the Seeds of *Sophora Tonkinensis* Gagnep. J. Agr. Food Chem. 68, 15015–15026. 10.1021/acs.jafc.0c06032 33285067

