# Supplementary material for: Uncommon Bis-Amide Matrine-type Alkaloids From Sophora alopecuroides With Anti-inflammatory Effects
Source: Front Chem. 2021 Sep 15;9:740421. doi: 10.3389/fchem.2021.740421 (PMC8479178; doi:10.3389/fchem.2021.740421)
Supplement: Supplementary file 1 [file DataSheet1.PDF]

Uncommon bis-amide matrine-type alkaloids from *Sophora alopecuroides* with anti-inflammatory effects

Ding Luo[1], Zhenchao Tu[1], Wenjing Yin[2], Chunlin Fan[1], Nenghua Chen[1], Zhongnan Wu[1], Yaolan Li[1*], Guocai Wang[1*], Yubo Zhang[1,2*]

[1]Institute of Traditional Chinese Medicine & Natural Products, Guangdong Province Key Laboratory of Pharmacodynamic Constituents of TCM and New Drugs Research, College of Pharmacy, Jinan University, Guangzhou 510632, People's Republic of China

[2]Guangdong Clinical Translational Center for Targeted Drug, Department of Pharmacology, School of Medicine, Jinan University, Guangzhou 510632, People's Republic of China

*** Correspondence:**
tliyl@jnu.edu.cn (Yaolan Li), twangguocai@jnu.edu.cn (Guocai Wang), ybzhang99@126.com (Yubo Zhang)

Content

Figure S1. UV spectrum of 1 in CH_3OH ... 4

Figure S2. CD spectrum of 1 in CH_3OH ... 4

Figure S3. IR spectrum of 1 (KBr disc) ... 5

Figure S4. HR-ESI-MS spectrum of 1 ... 5

Figure S5. ^1H NMR (600 M, $CDCl_3$) spectrum of 1 ... 6

Figure S6. ^{13}C NMR (150 MHz, $CDCl_3$) spectrum of 1 ... 6

Figure S7. DEPT-135 spectrum of 1 in$CDCl_3$ ... 7

Figure S8. ^1H-^1H COSY spectrum of 1 in$CDCl_3$ ... 7

Figure S9. HSQC spectrum of 1 in$CDCl_3$ ... 8

Figure S10. HMBC spectrum of 1 in$CDCl_3$ ... 8

Figure S11. NOESY spectrum of 1 in$CDCl_3$ ... 9

Figure S12. UV spectrum of 2 in CH_3OH ... 9

Figure S13. CD spectrum of 2 in CH_3OH ... 10

Figure S14. IR spectrum of 2 (KBr disc) ... 10

Figure S15. HR-ESI-MS spectrum of 2 ... 11

Figure S16. ^1H NMR (600 M, $CDCl_3$) spectrum of 2 ... 11

Figure S17. ^{13}C NMR (150 MHz, $CDCl_3$) spectrum of 2 ... 12

Figure S18. DEPT-135 spectrum of 2 in$CDCl_3$ ... 12

Figure S19. ^1H-^1H COSY spectrum of 2 in$CDCl_3$ ... 13

Figure S20. HSQC spectrum of 2 in$CDCl_3$ ... 13

Figure S21. HMBC spectrum of 2 in$CDCl_3$ ... 14

Figure S22. NOESY spectrum of 2 in$CDCl_3$ ... 14

Figure S23. UV spectrum of 3 in CH_3OH ... 15

Figure S24. CD spectrum of 3 in CH_3OH ... 15

Figure S25. IR spectrum of 3 (KBr disc) ... 15

Figure S26. HR-ESI-MS spectrum of 3 ... 16

Figure S27. ^1H NMR (600 M, $CDCl_3$) spectrum of 3 ... 16

Figure S28. ^{13}C NMR (150 MHz, $CDCl_3$) spectrum of 3 ... 17

Figure S29. DEPT-135 spectrum of 3 in$CDCl_3$ ... 17

Figure S30. ^1H-^1H COSY spectrum of 3 in$CDCl_3$ ... 18

Figure S31. HSQC spectrum of 3 in$CDCl_3$ 18

Figure S32. HMBC spectrum of 3 in$CDCl_3$ 19

Figure S33. NOESY spectrum of 3 in$CDCl_3$ 19

Figure S34. UV spectrum of 4 in CH_3OH 20

Figure S35. CD spectrum of 4 in CH_3OH 20

Figure S36. IR spectrum of 4 (KBr disc) 21

Figure S37. HR-ESI-MS spectrum of 4 21

Figure S38. ^{1}H NMR (600 M, $CDCl_3$) spectrum of 4 22

Figure S39. ^{13}C NMR (150 MHz, $CDCl_3$) spectrum of 4 22

Figure S40. DEPT-135 spectrum of 4 in$CDCl_3$ 23

Figure S41. ^{1}H-^{1}H COSY spectrum of 4 in$CDCl_3$ 23

Figure S42. HSQC spectrum of 4 in$CDCl_3$ 24

Figure S43. HMBC spectrum of 4 in$CDCl_3$ 24

Figure S44. NOESY spectrum of 4 in$CDCl_3$ 25

Figure S45. The predominant low-energy conformers of 2 at the B3LYP/6-31+G(d, p) level by Gaussian 09 program. 26

Table S1. Gibbs free energies and equilibrium populations of low-energy conformers of 2 26

Table S2. Standard orientations of Conformers 2A−2B. 27

Figure S46. The predominant low-energy conformer of 4 at the B3LYP/6-31+G(d, p) level by Gaussian 09 program. 29

Table S3. Gibbs free energies and equilibrium populations of low-energy conformer of 4 29

Table S4. Standard orientations of Conformer 4. 30

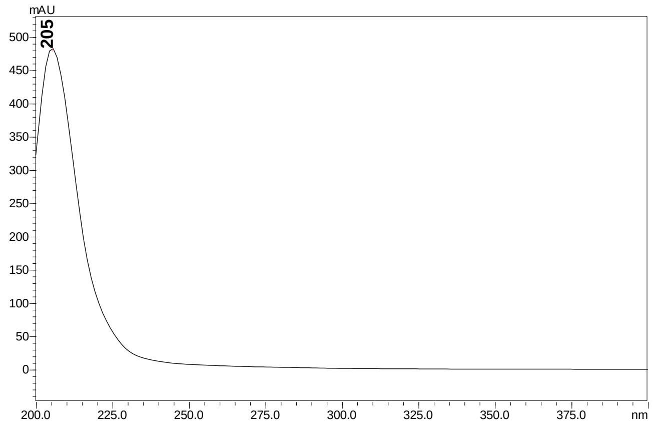

Figure S1. UV spectrum of 1 in CH_3OH

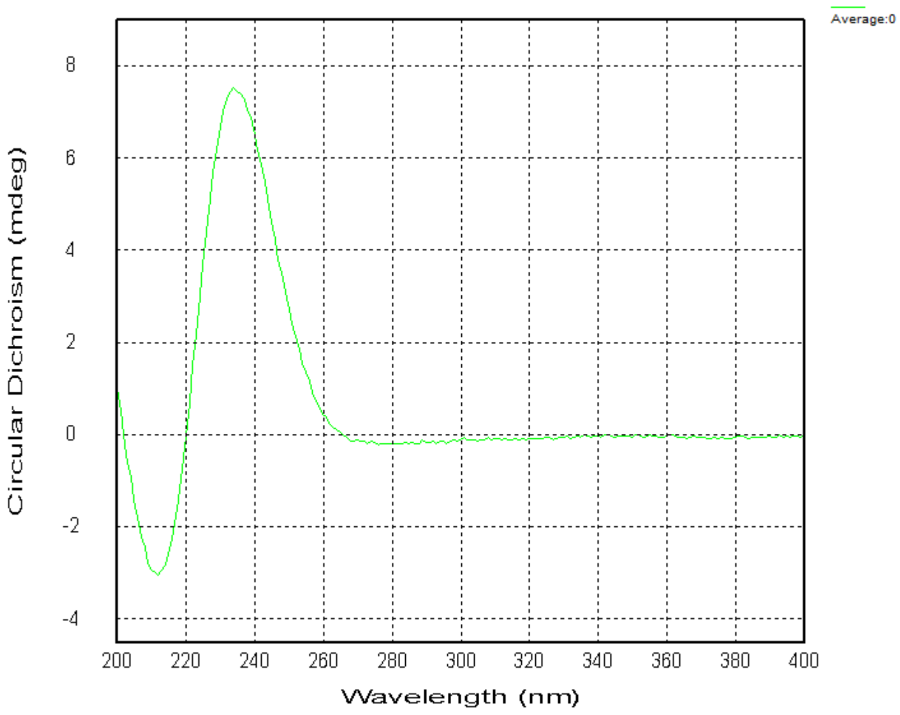

Figure S2. CD spectrum of 1 in CH_3OH

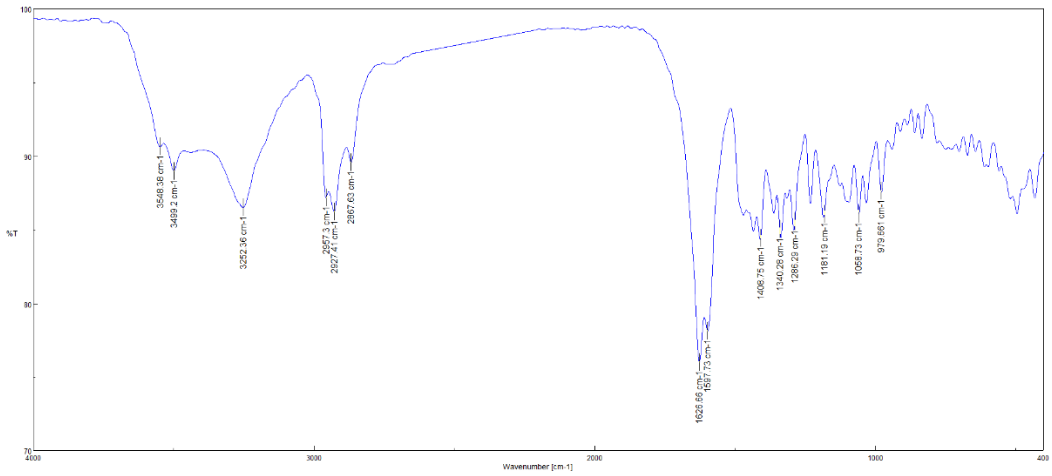

Figure S3. IR spectrum of 1 (KBr disc)

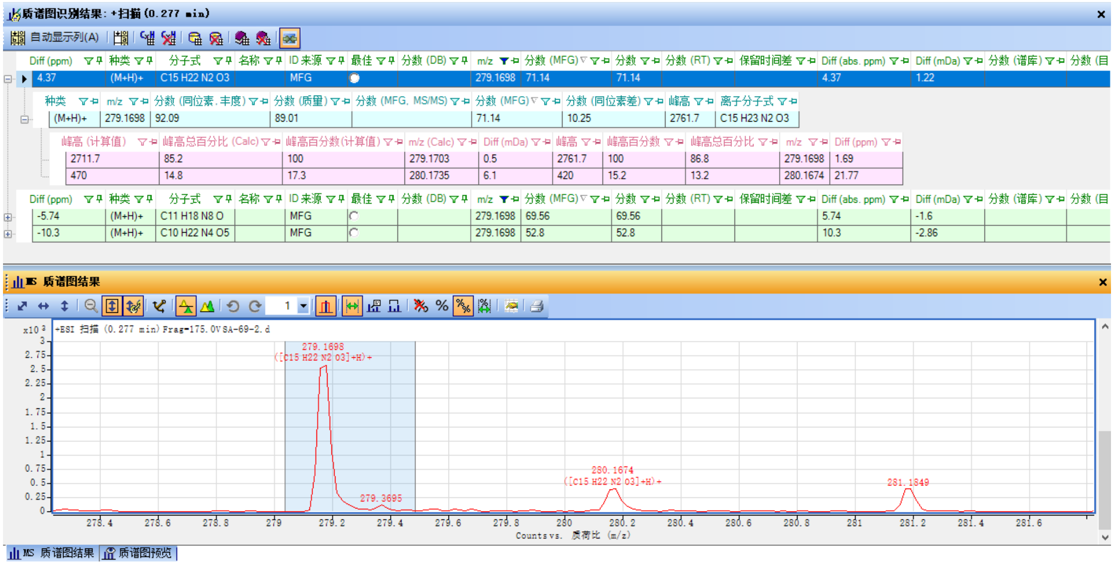

Figure S4. HR-ESI-MS spectrum of 1

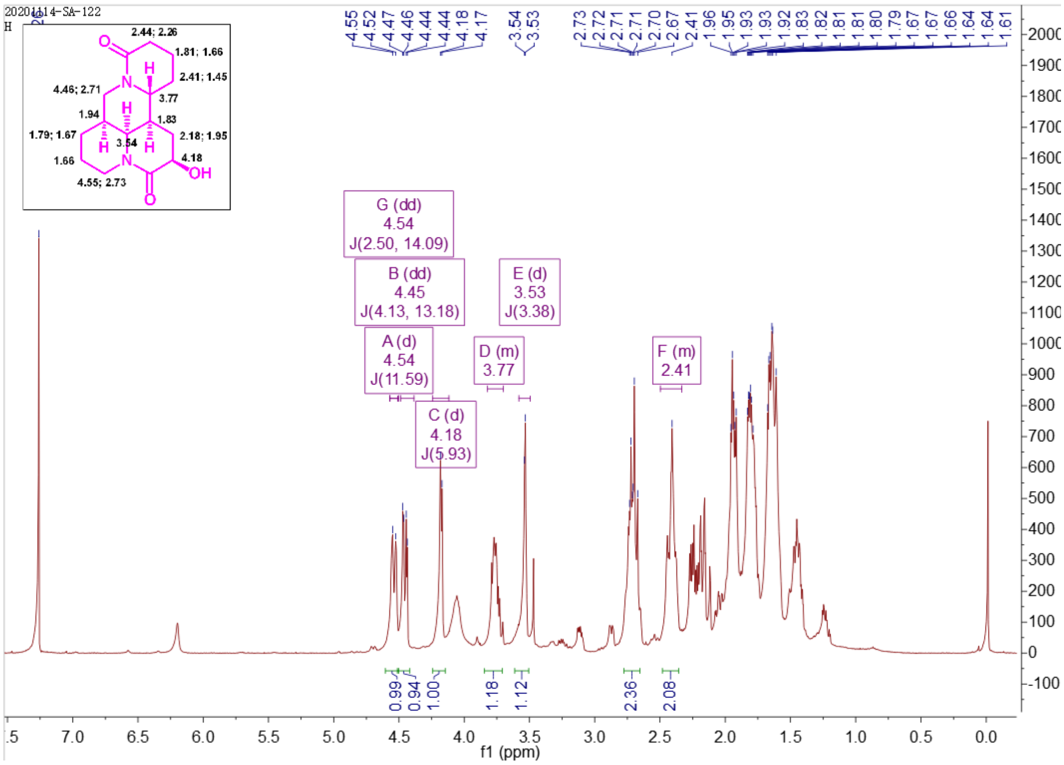

Figure S5. [1]**H NMR (600 M, CDCl₃) spectrum of 1**

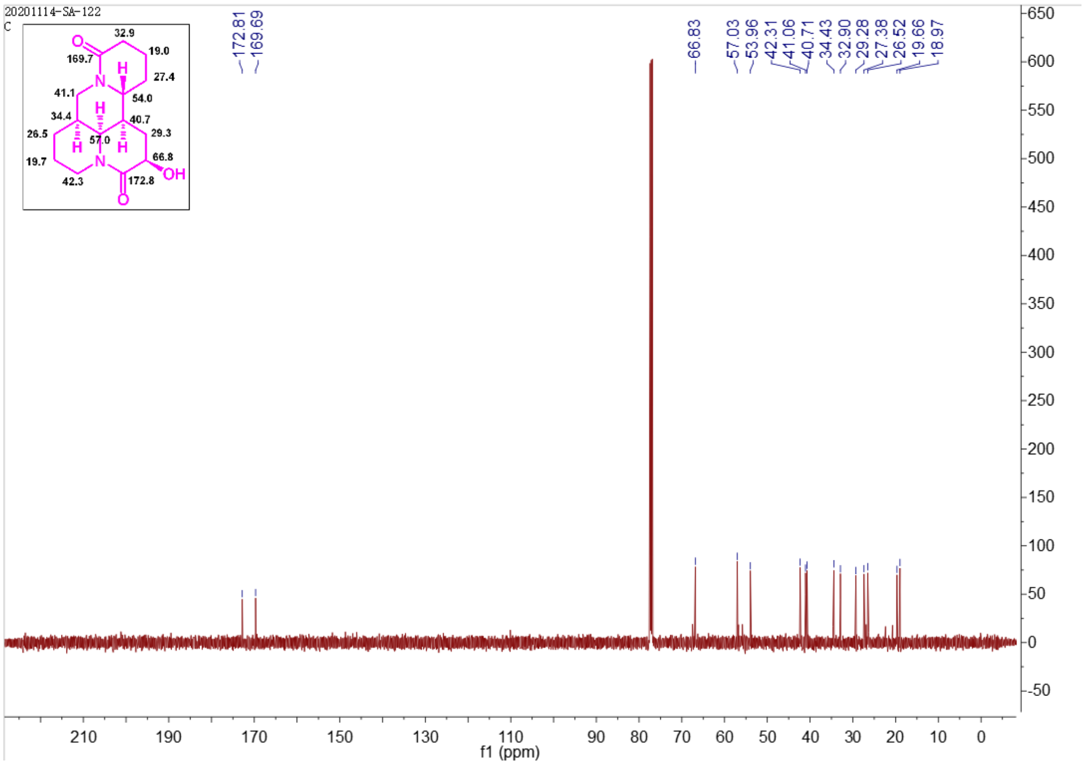

Figure S6. [13]**C NMR (150 MHz, CDCl₃) spectrum of 1**

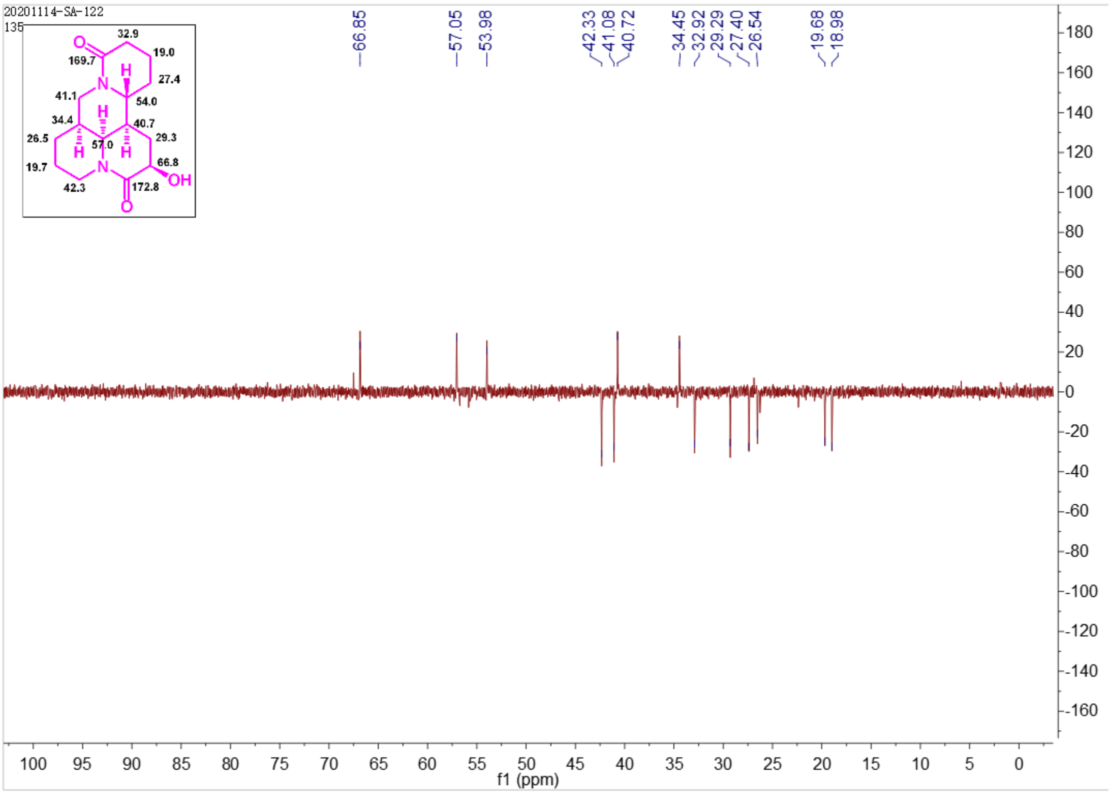

Figure S7. DEPT-135 spectrum of 1 inCDCl3

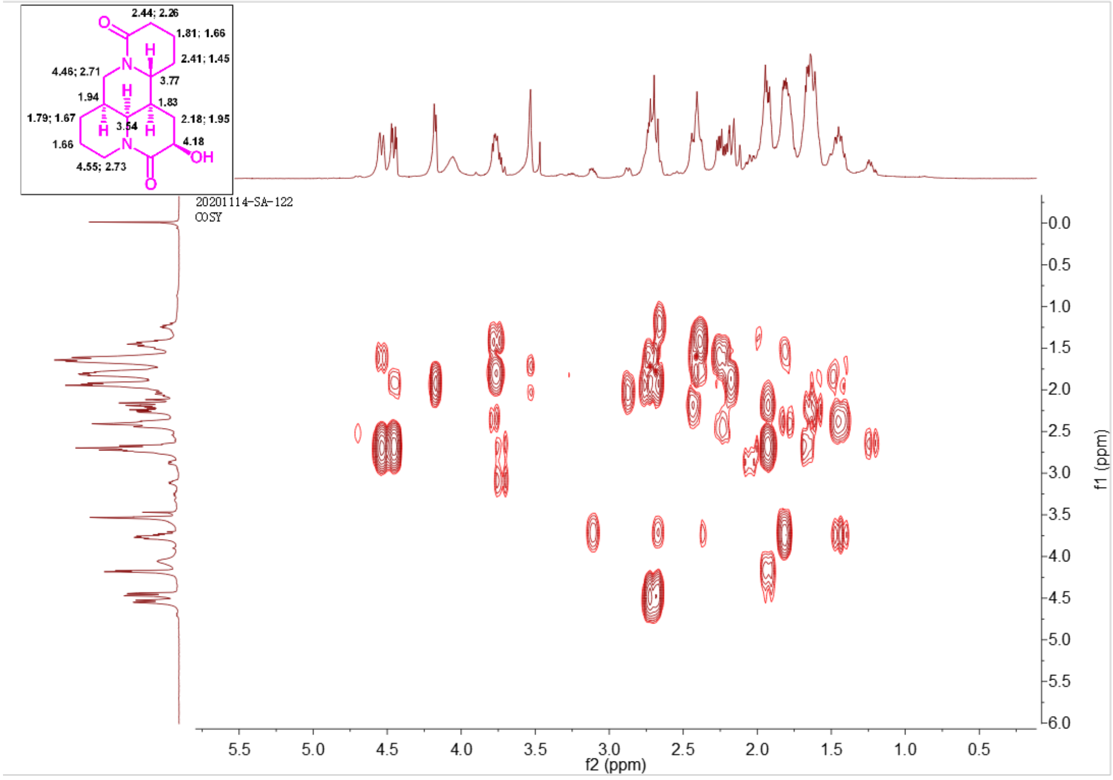

Figure S8. ^{1}H-^{1}H COSY spectrum of 1 inCDCl3

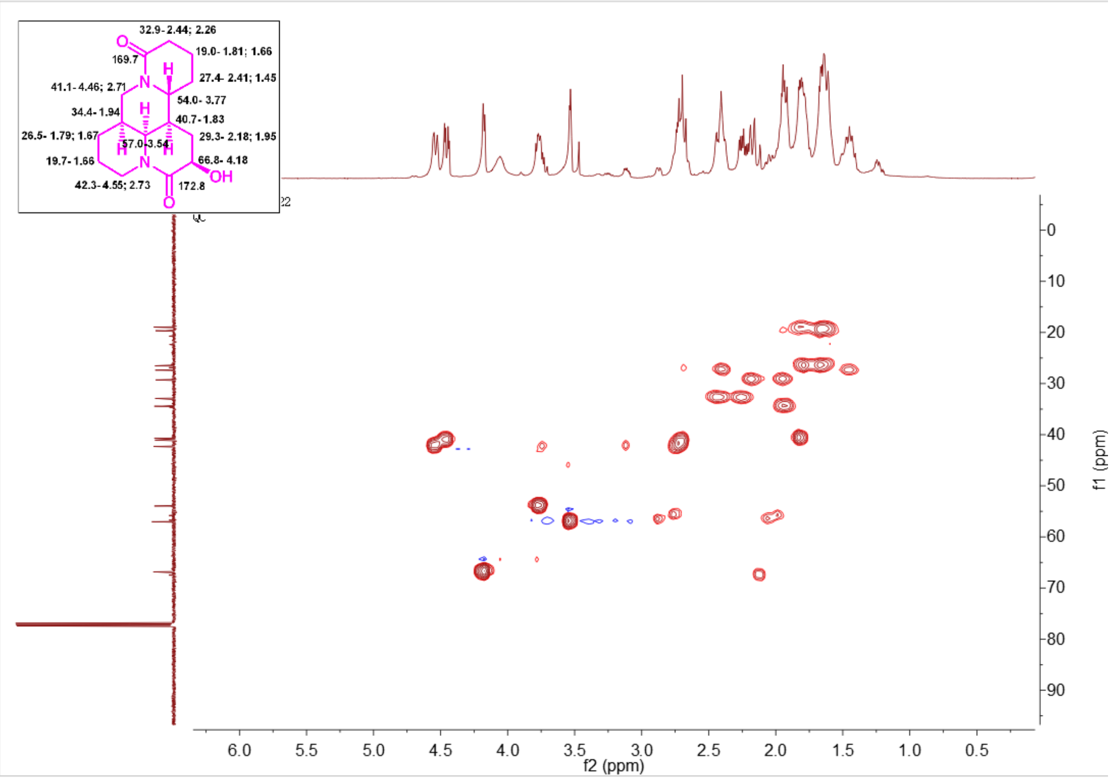

Figure S9. HSQC spectrum of 1 inCDCl₃

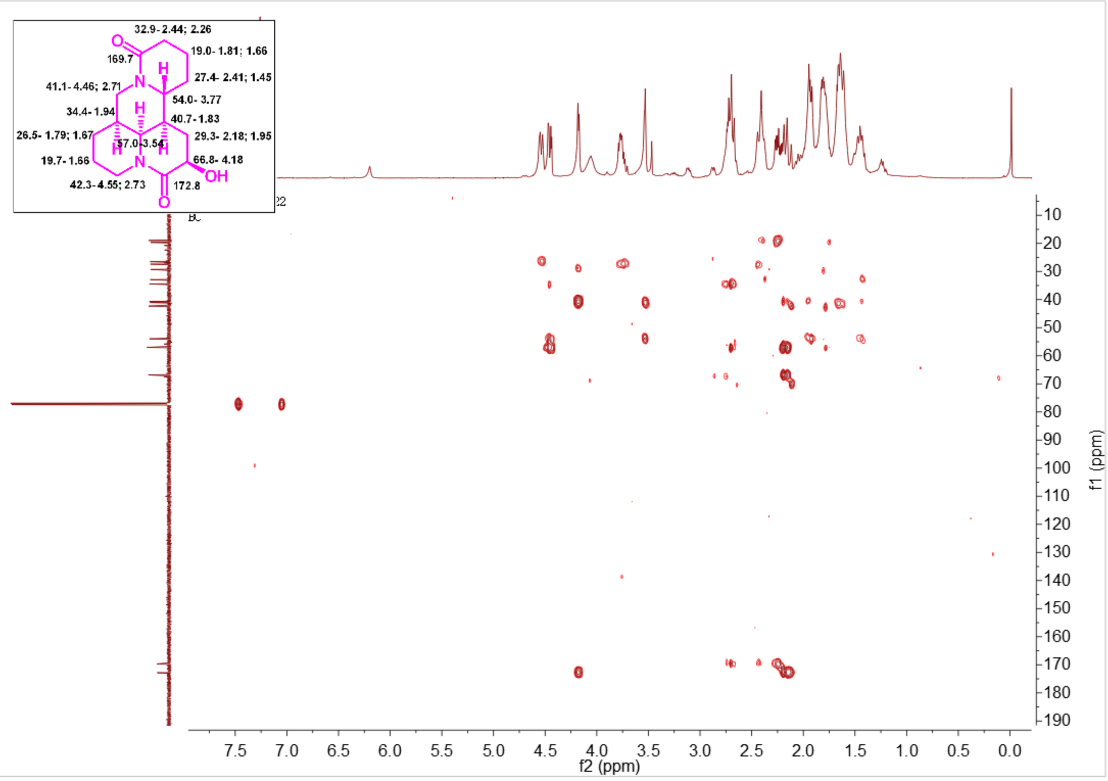

Figure S10. HMBC spectrum of 1 inCDCl₃

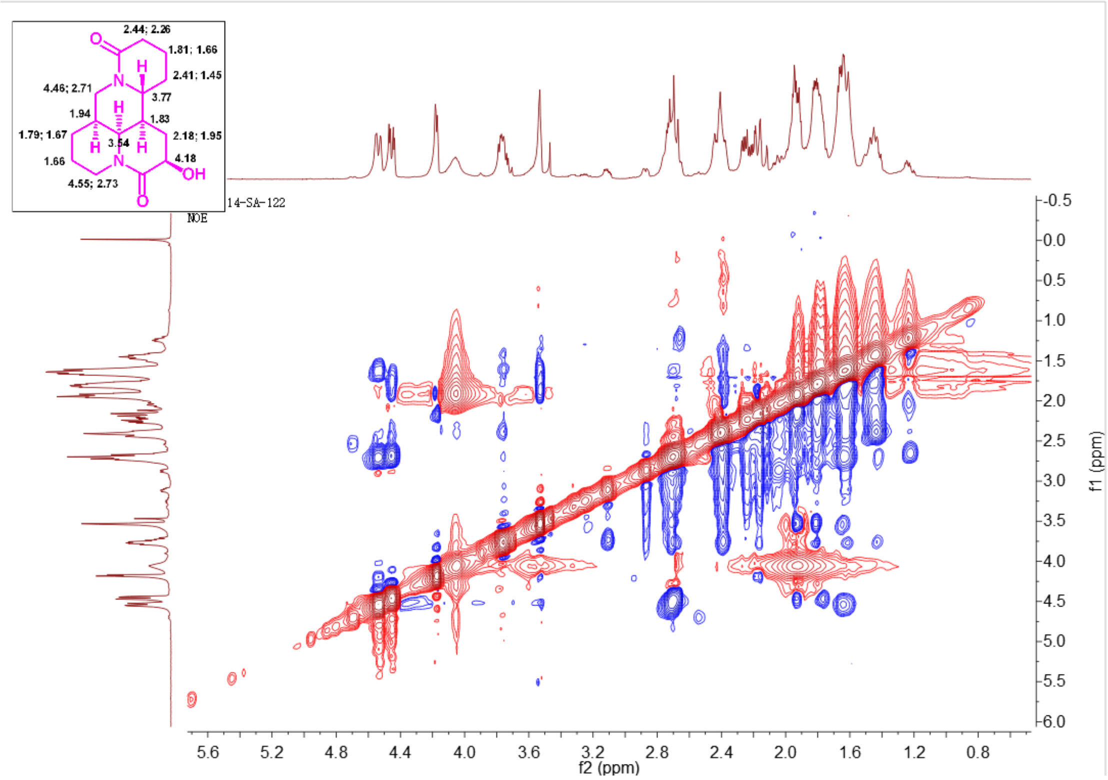

Figure S11. NOESY spectrum of 1 inCDCl₃

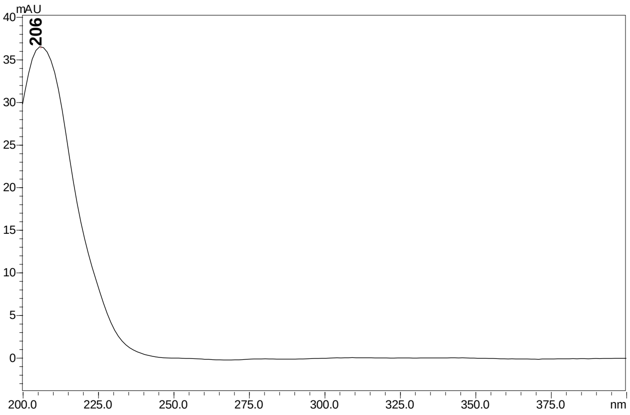

Figure S12. UV spectrum of 2 in CH_3OH

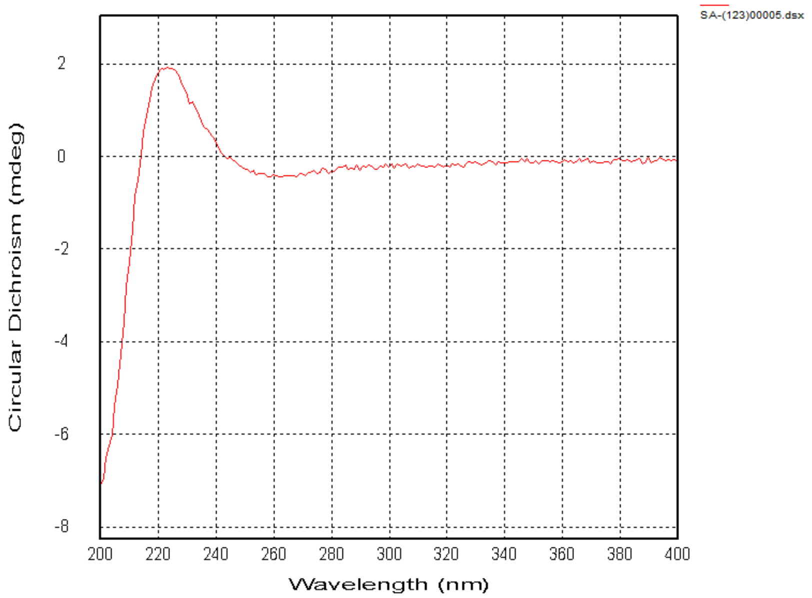

Figure S13. CD spectrum of 2 in CH_3OH

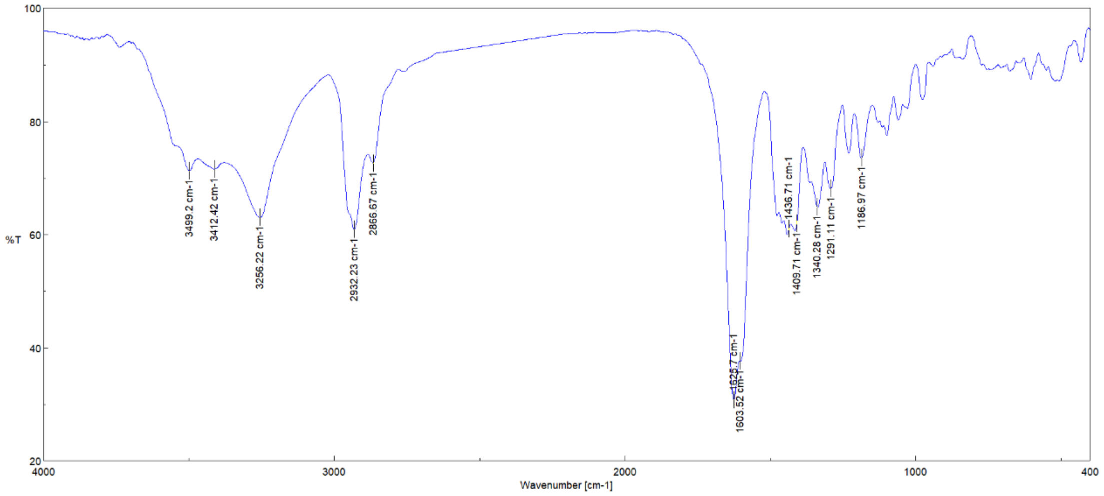

Figure S14. IR spectrum of 2 (KBr disc)

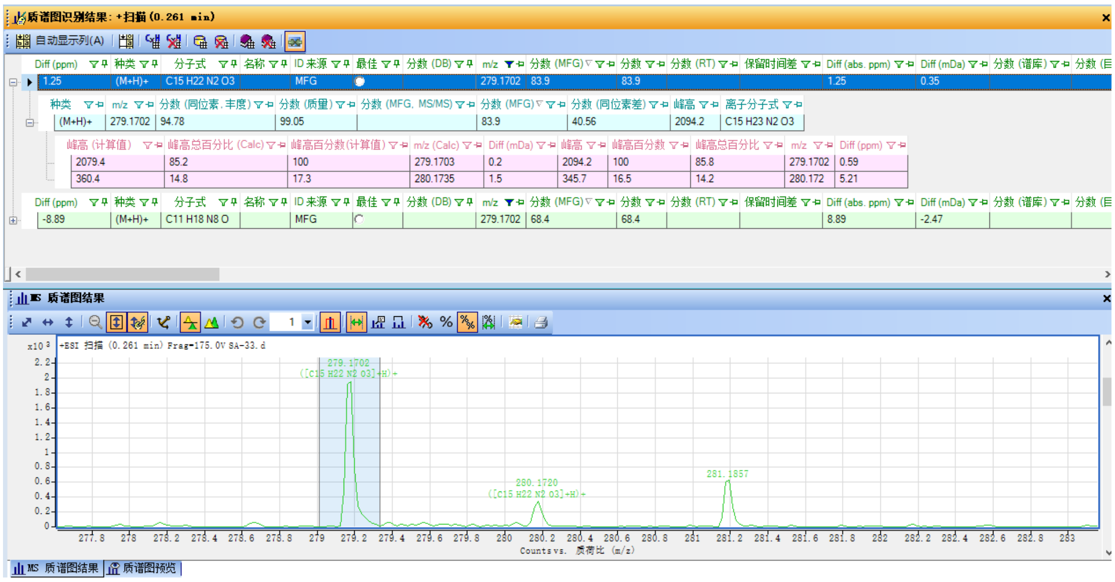

Figure S15. HR-ESI-MS spectrum of 2

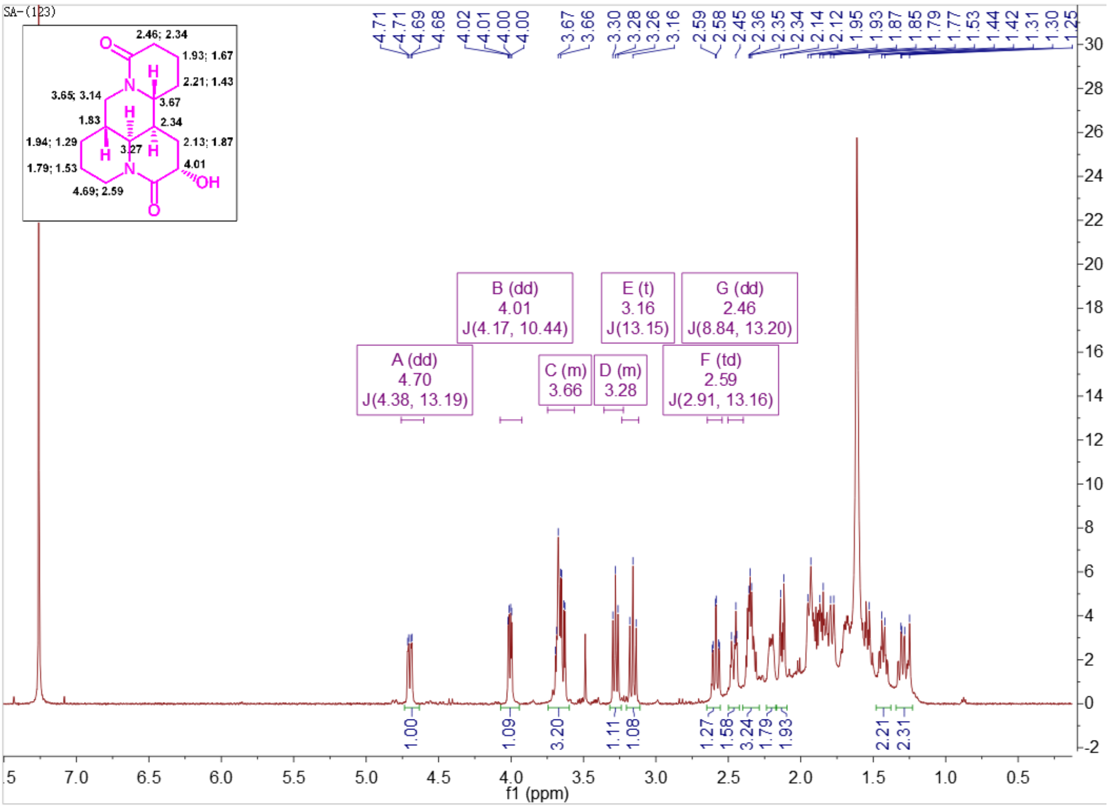

Figure S16. ^{1}H NMR (600 M, $CDCl_3$) spectrum of 2

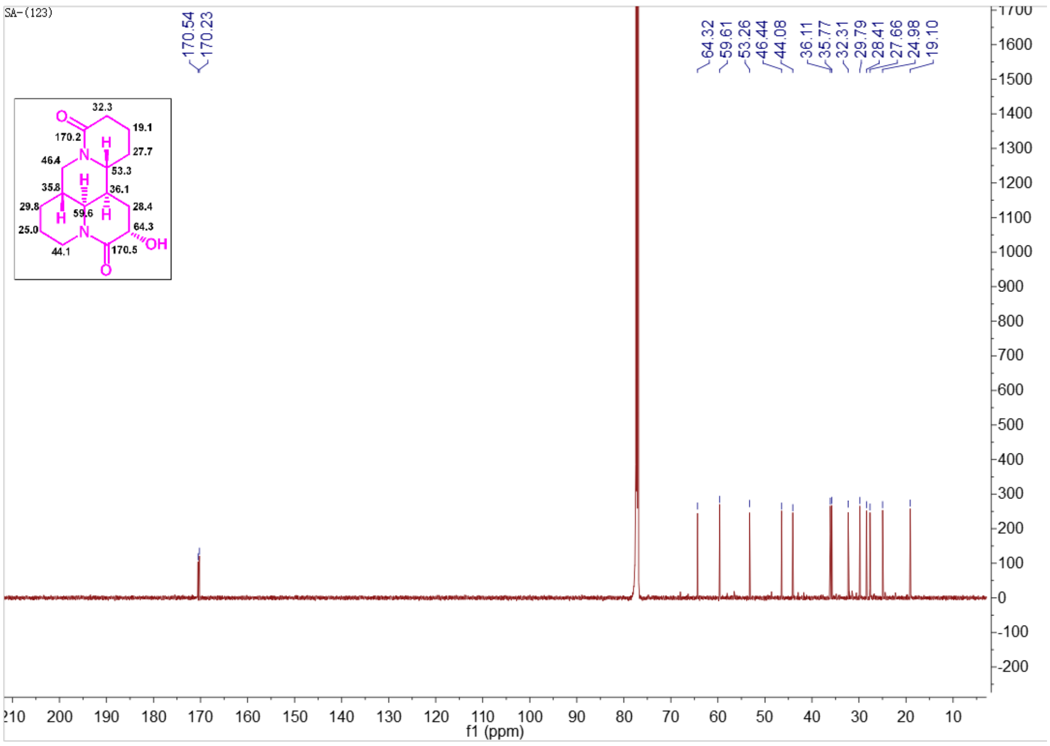

Figure S17. ^{13}C NMR (150 MHz, CDCl₃) spectrum of 2

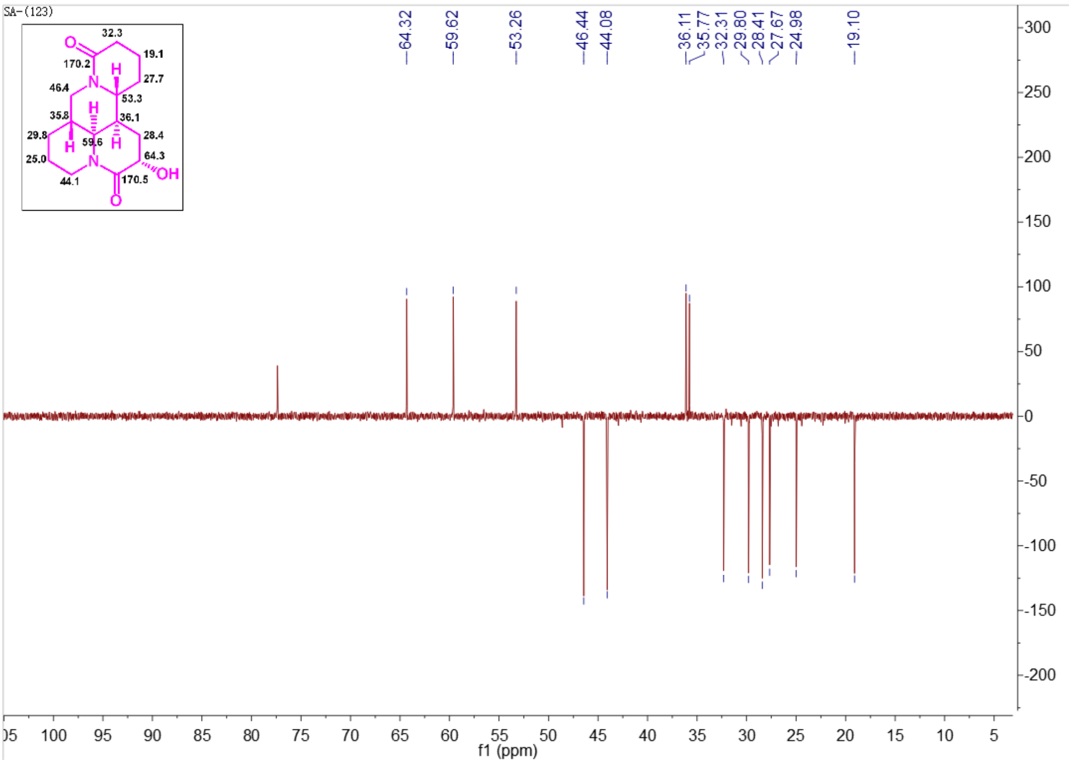

Figure S18. DEPT-135 spectrum of 2 inCDCl₃

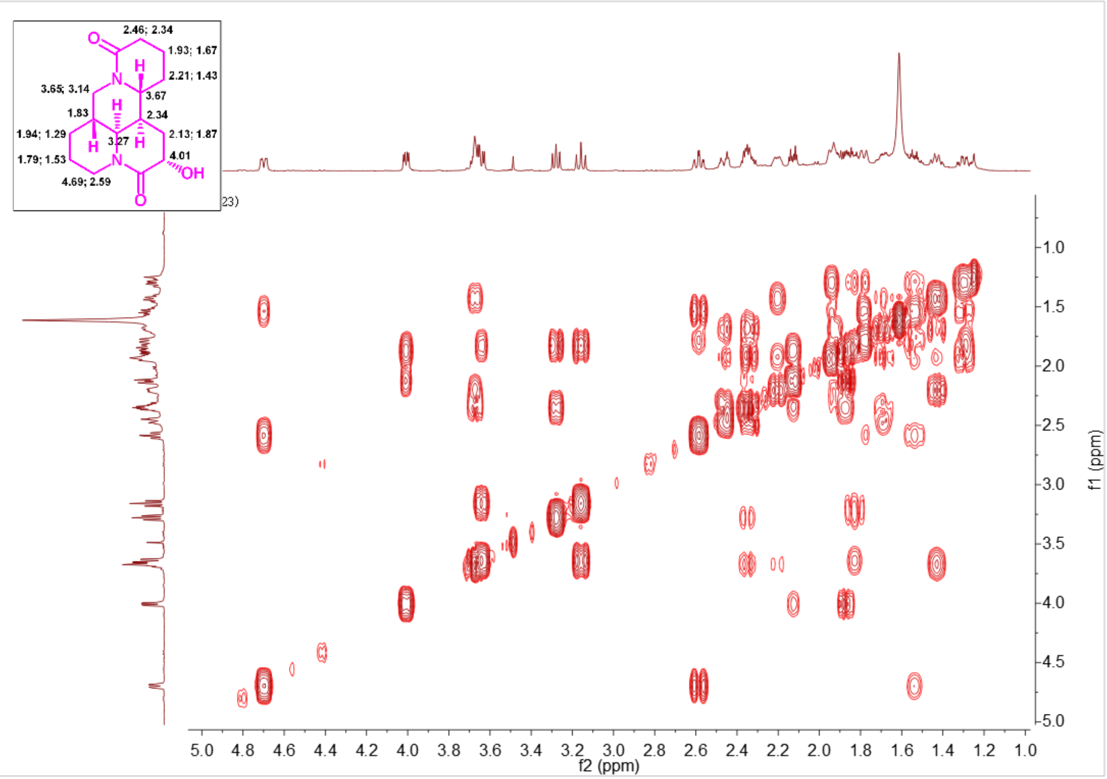

Figure S19. ^{1}H-^{1}H COSY spectrum of 2 inCDCl3

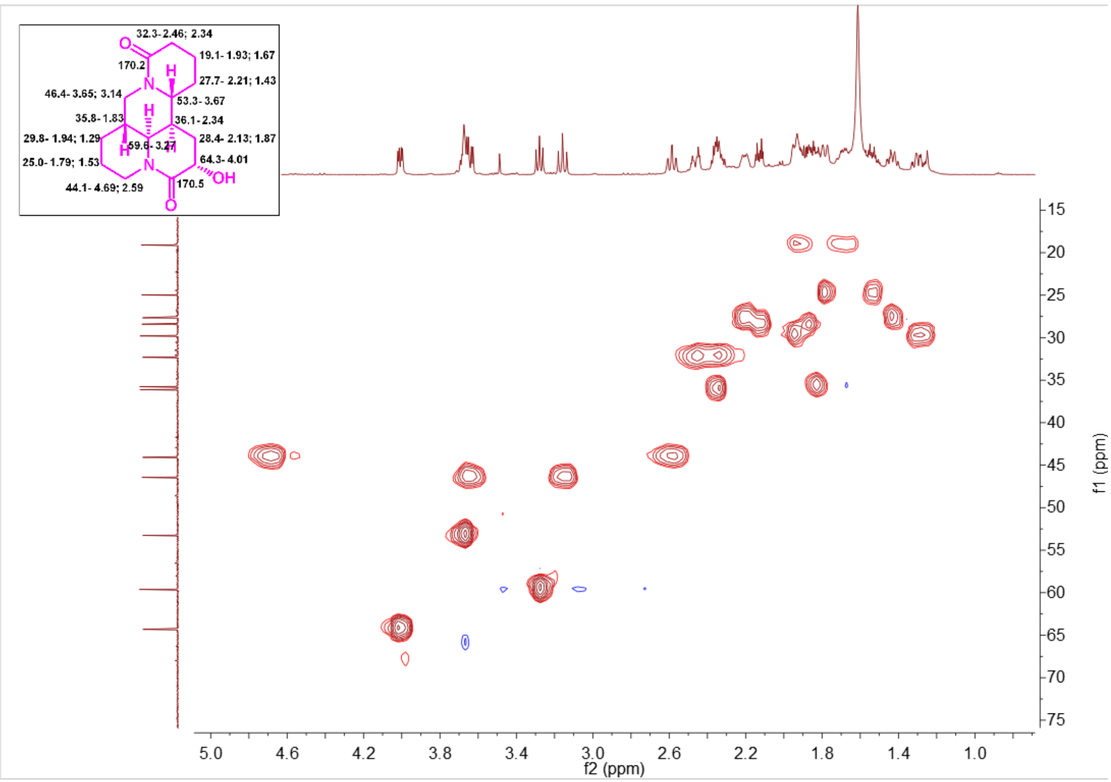

Figure S20. HSQC spectrum of 2 inCDCl3

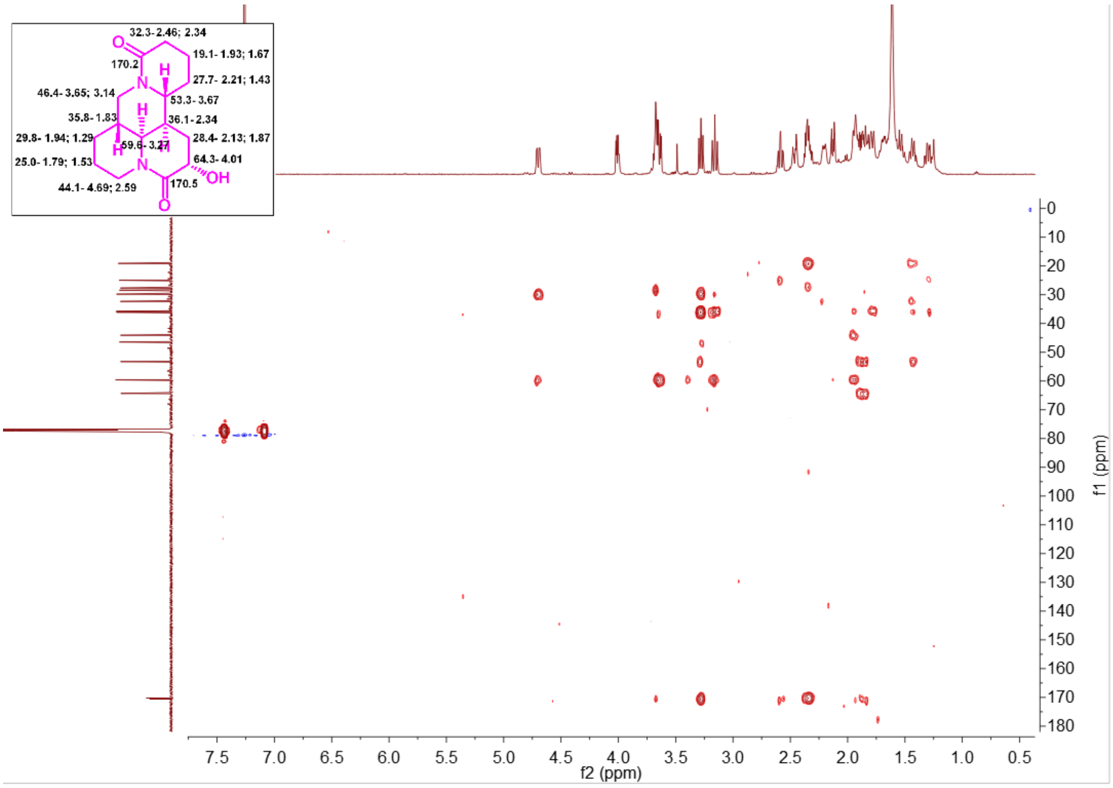

Figure S21. HMBC spectrum of 2 inCDCl3

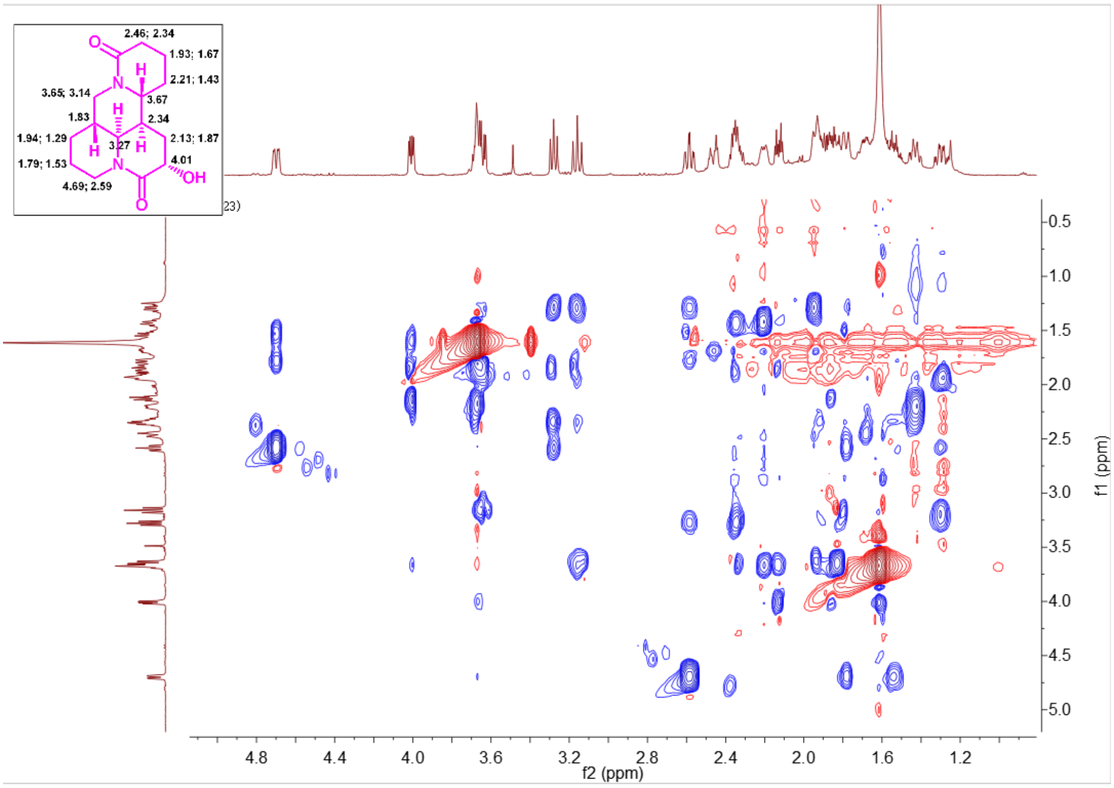

Figure S22. NOESY spectrum of 2 inCDCl3

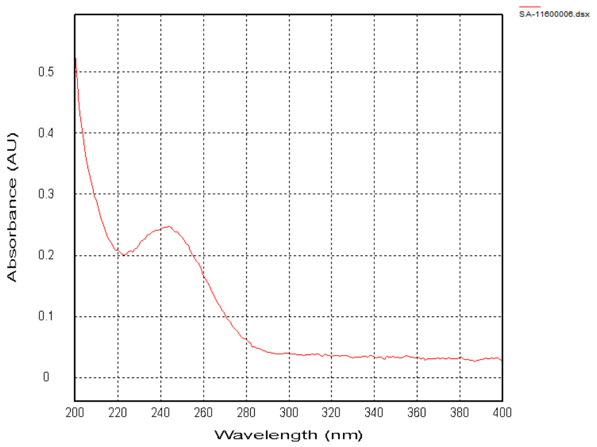

Figure S23. UV spectrum of 3 in CH_3OH

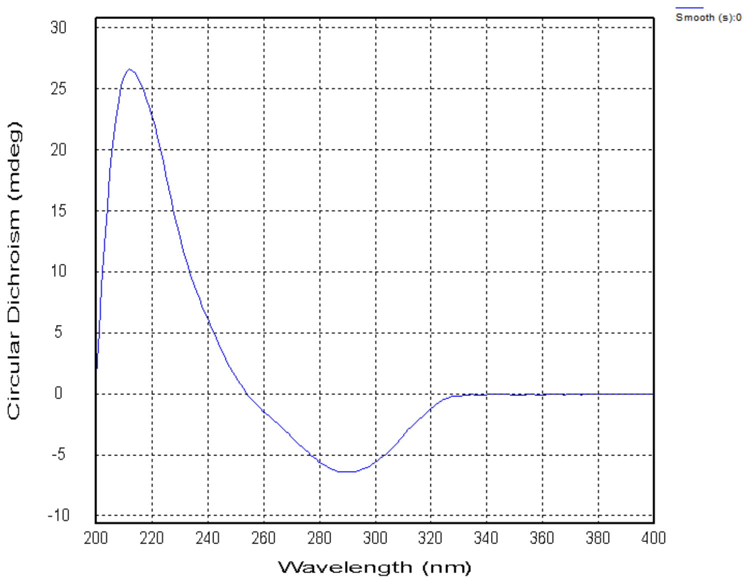

Figure S24. CD spectrum of 3 in CH_3OH

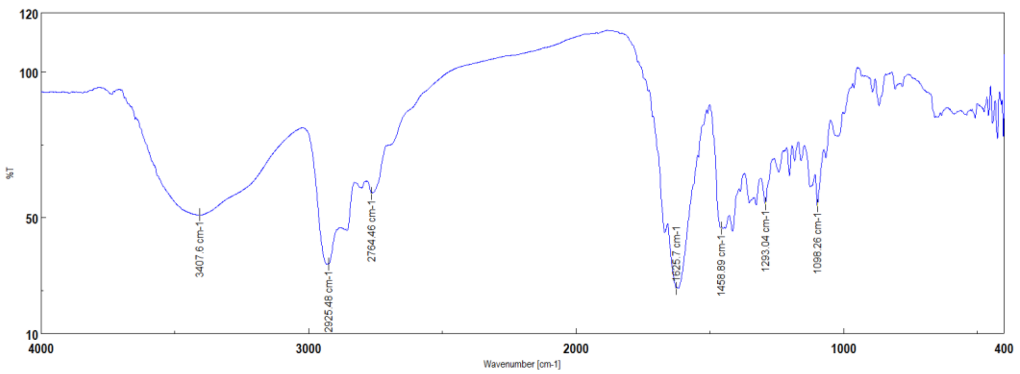

Figure S25. IR spectrum of 3 (KBr disc)

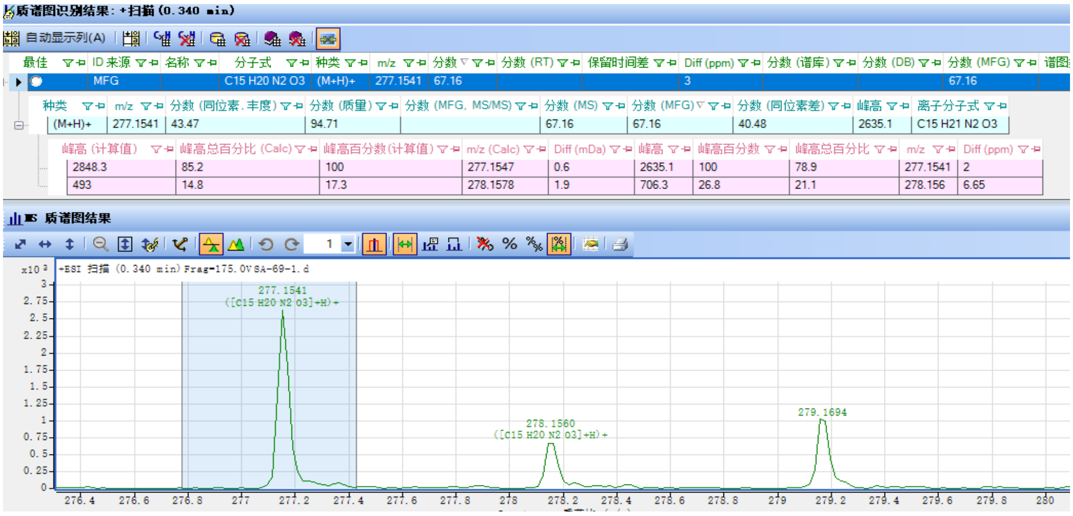

Figure S26. HR-ESI-MS spectrum of 3

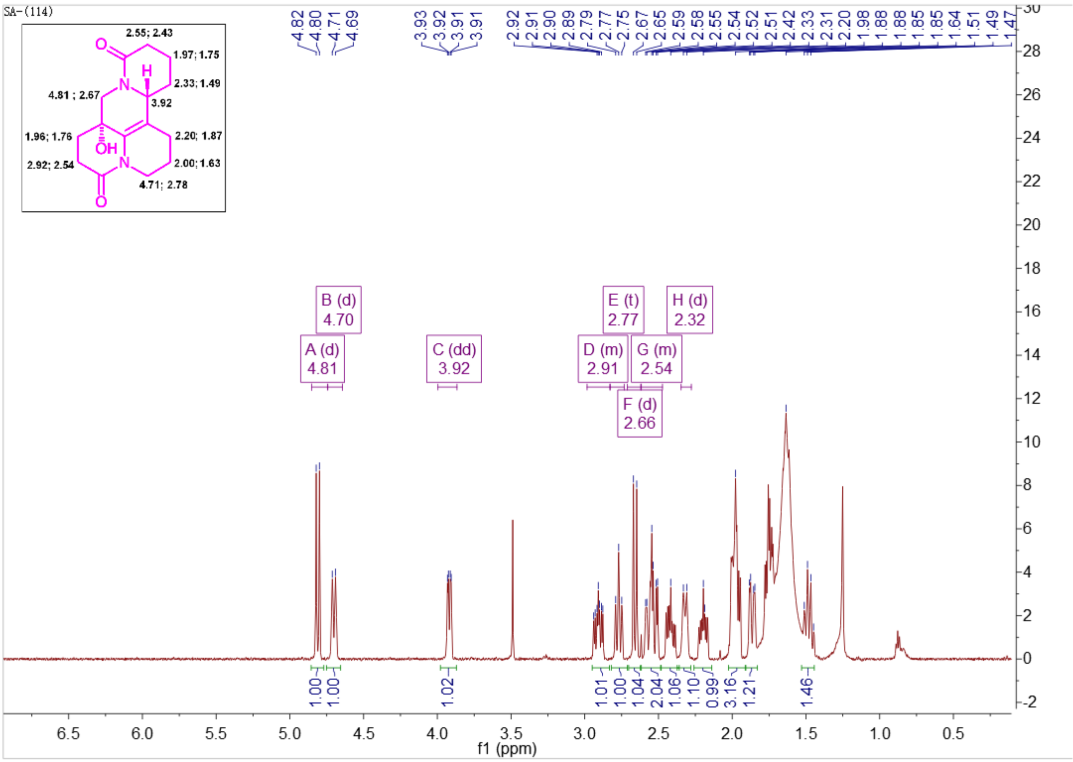

Figure S27. ^{1}H NMR (600 M, $CDCl_3$) spectrum of 3

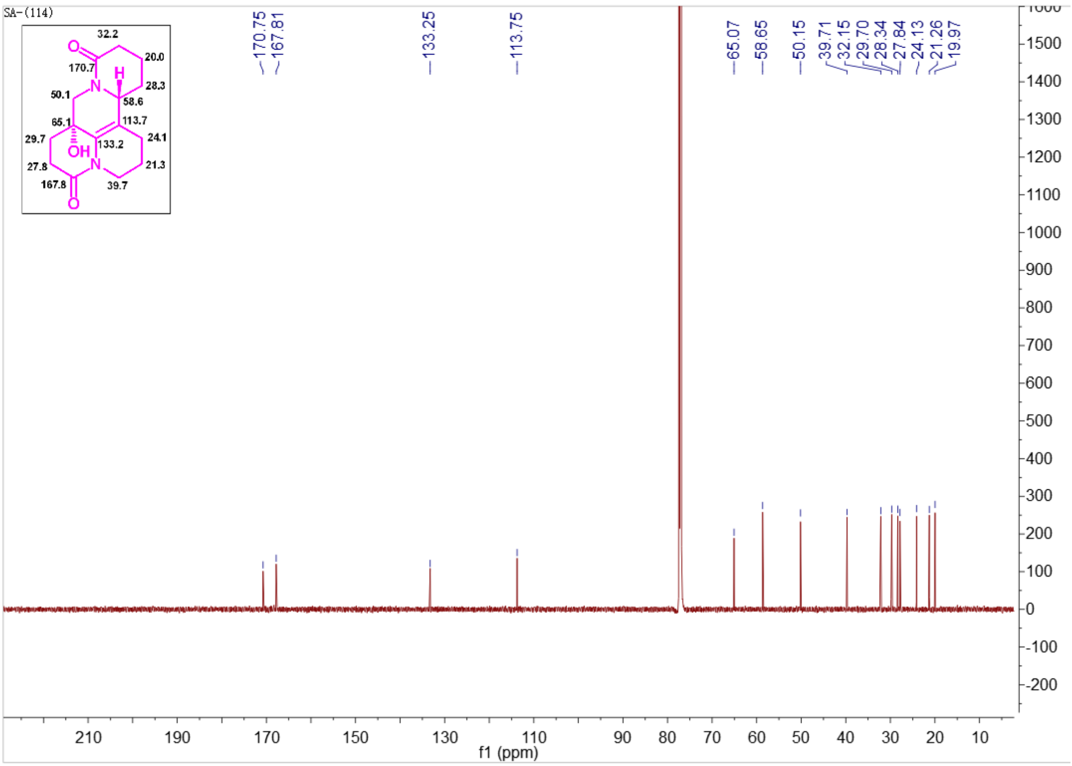

Figure S28. ^{13}C NMR (150 MHz, $CDCl_3$) spectrum of 3

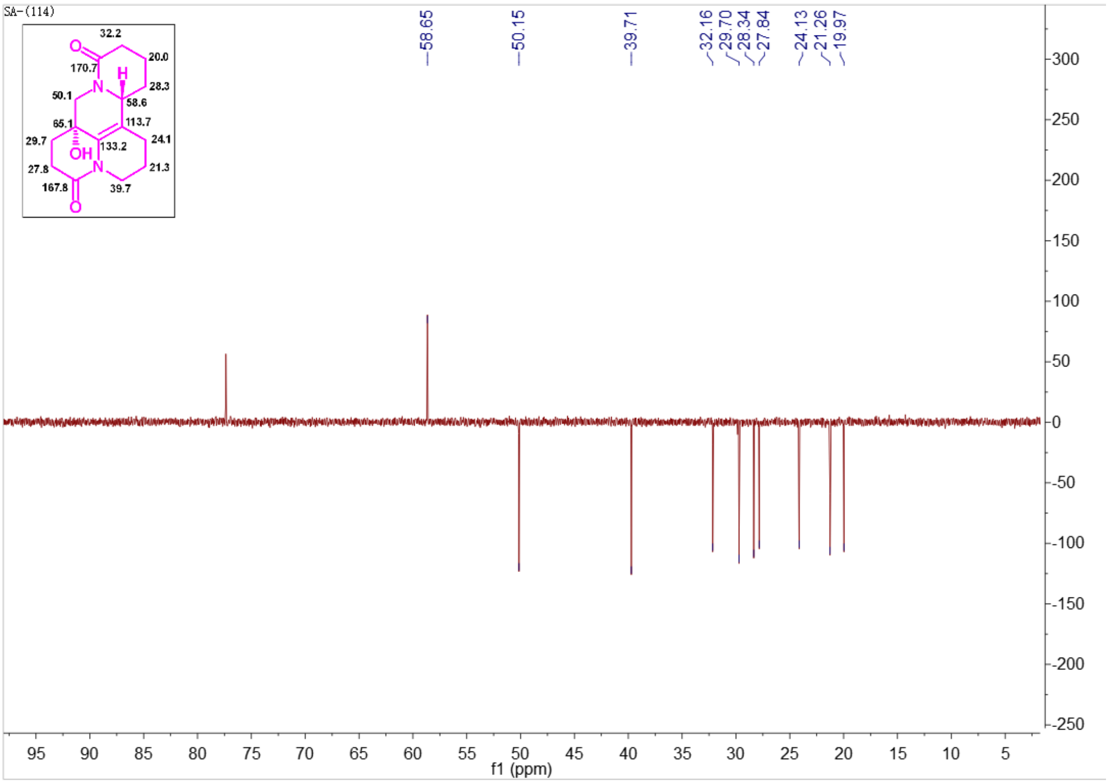

Figure S29. DEPT-135 spectrum of 3 in$CDCl_3$

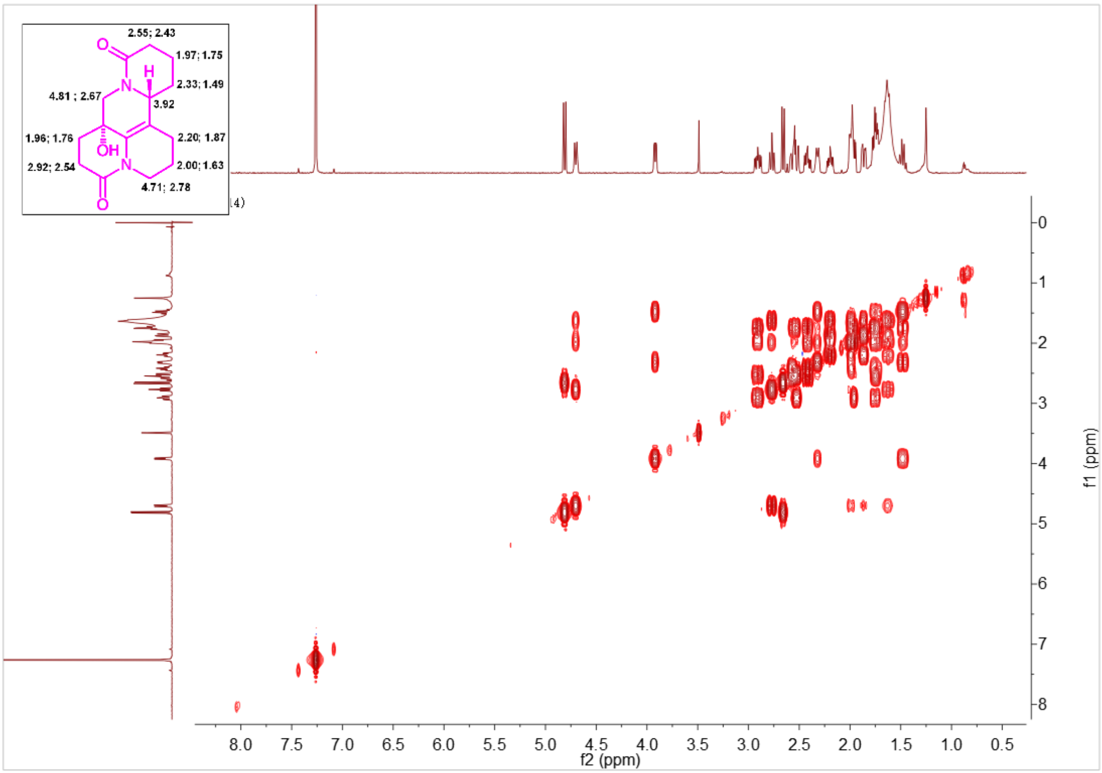

Figure S30. **^{1}H-^{1}H COSY spectrum of 3 inCDCl₃**

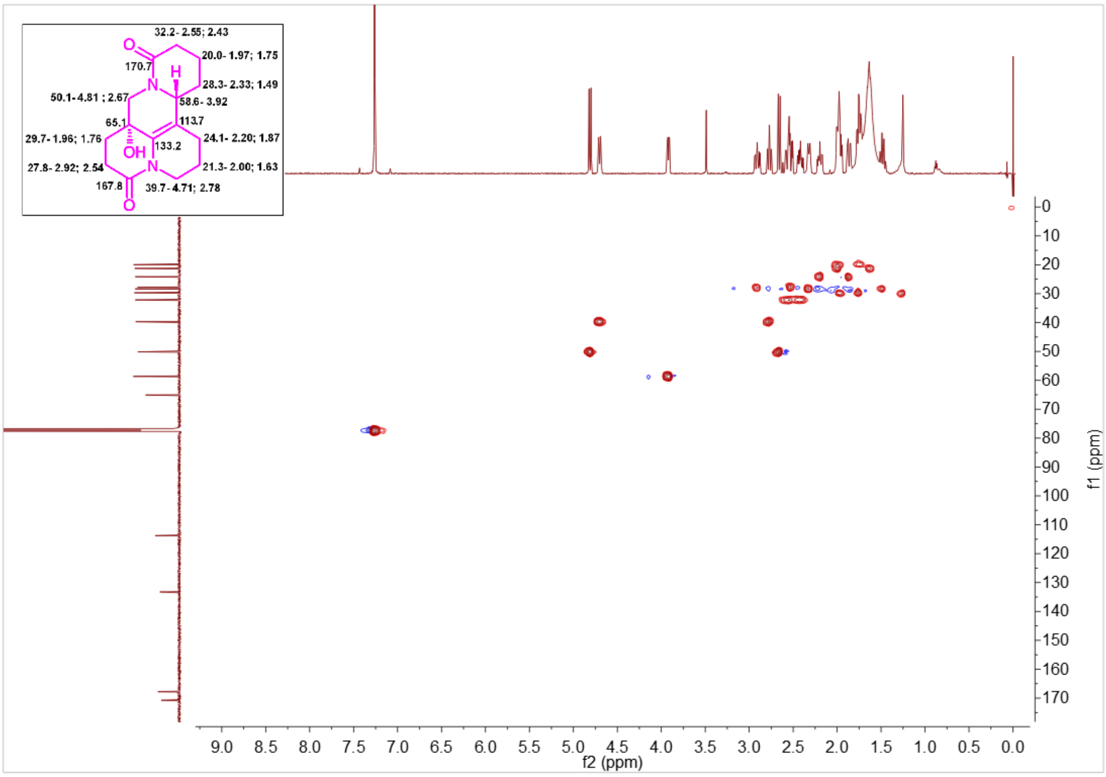

Figure S31. HSQC spectrum of 3 inCDCl₃

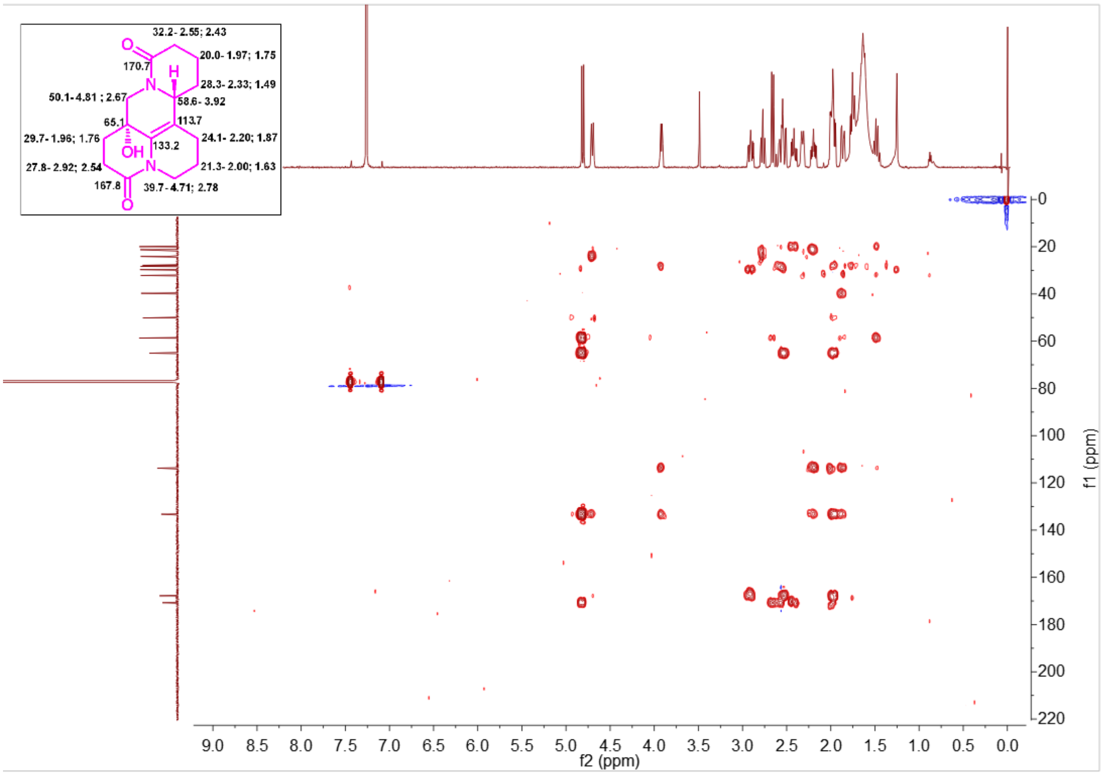

Figure S32. HMBC spectrum of 3 inCDCl3

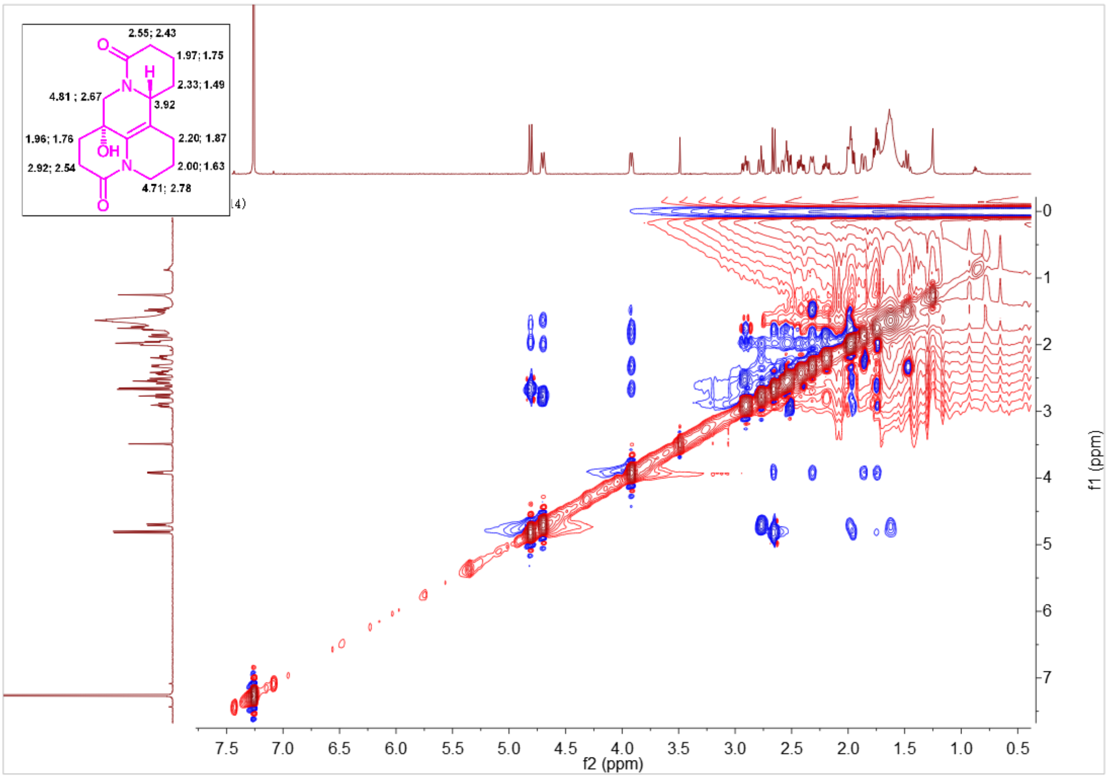

Figure S33. NOESY spectrum of 3 inCDCl3

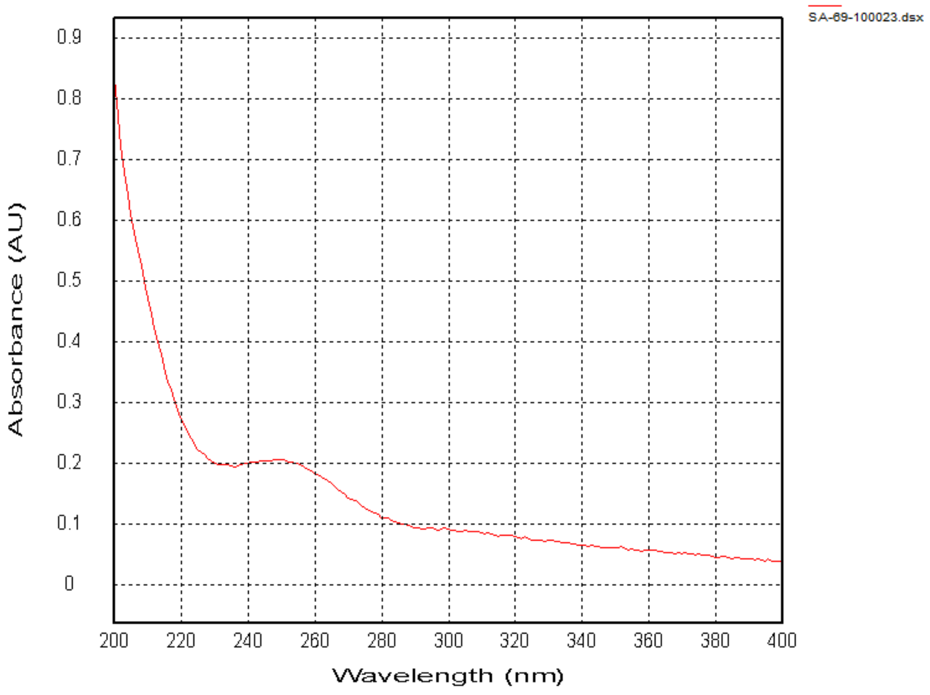

Figure S34. UV spectrum of 4 in CH_3OH

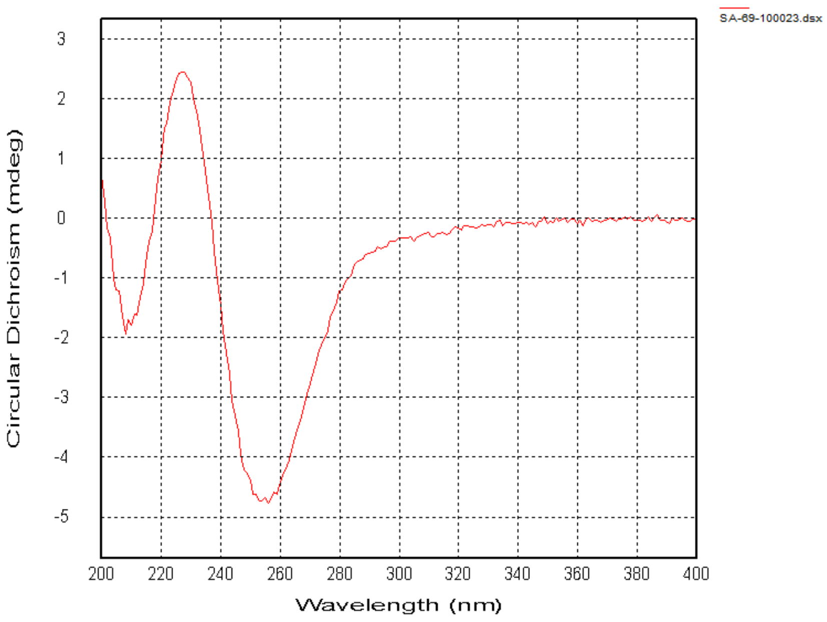

Figure S35. CD spectrum of 4 in CH_3OH

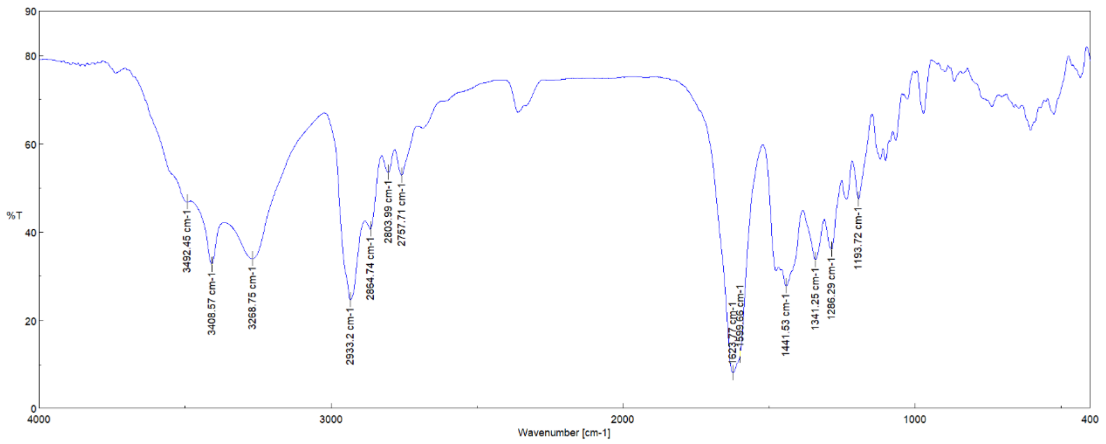

Figure S36. IR spectrum of 4 (KBr disc)

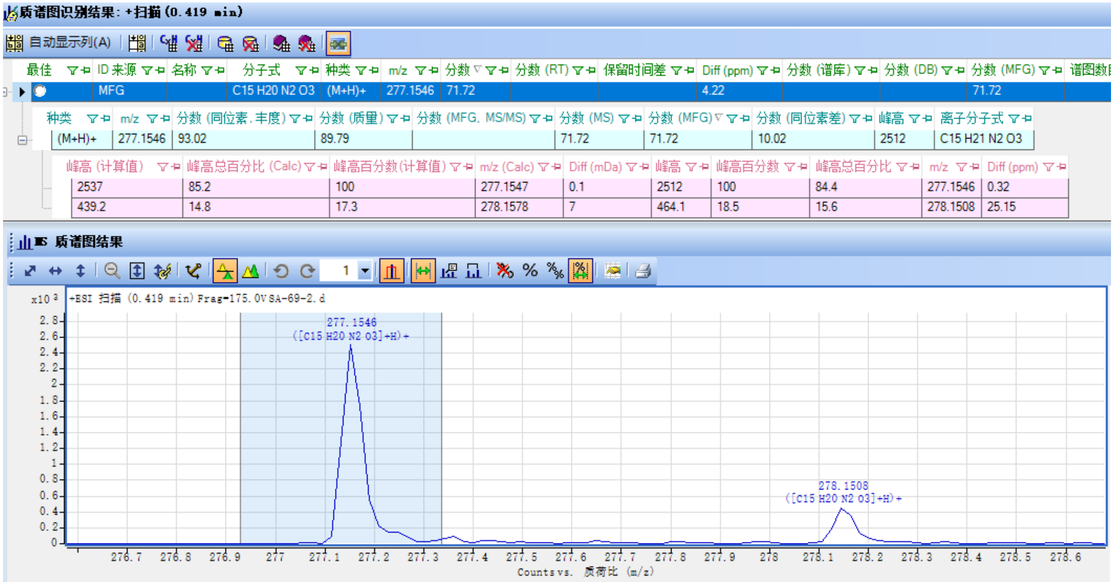

Figure S37. HR-ESI-MS spectrum of 4

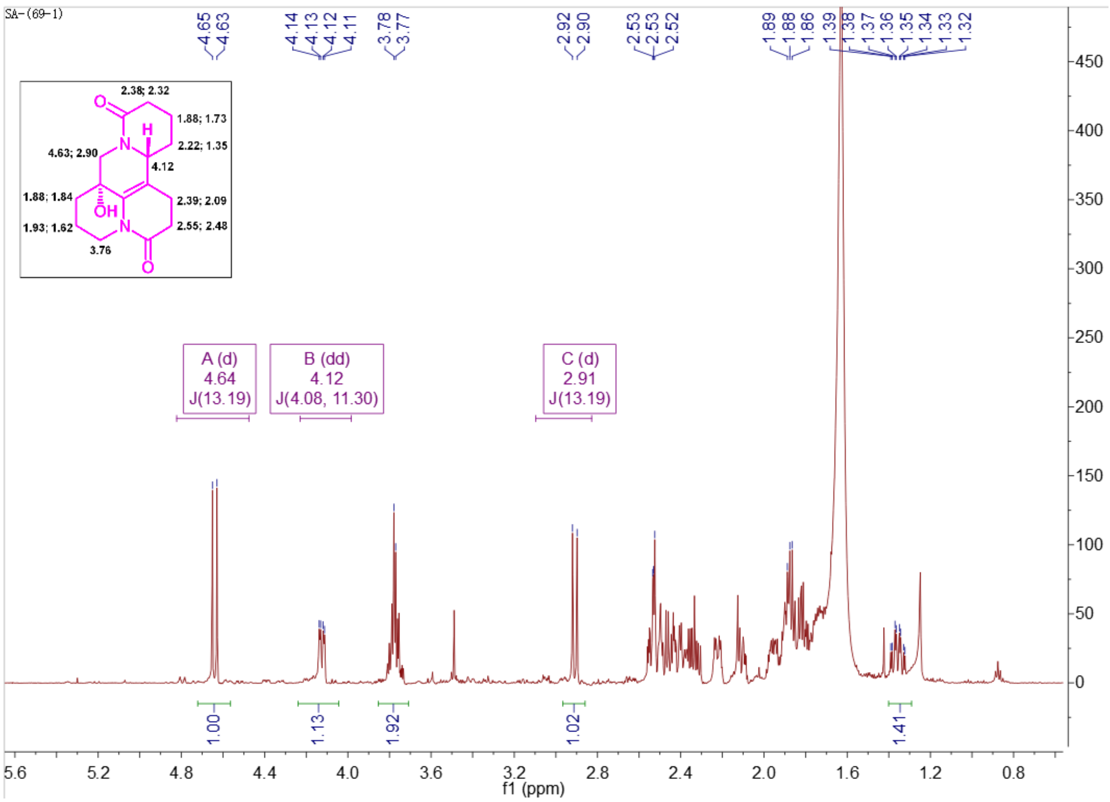

Figure S38. ^{1}**H NMR (600 M,** $CDCl_3$**) spectrum of 4**

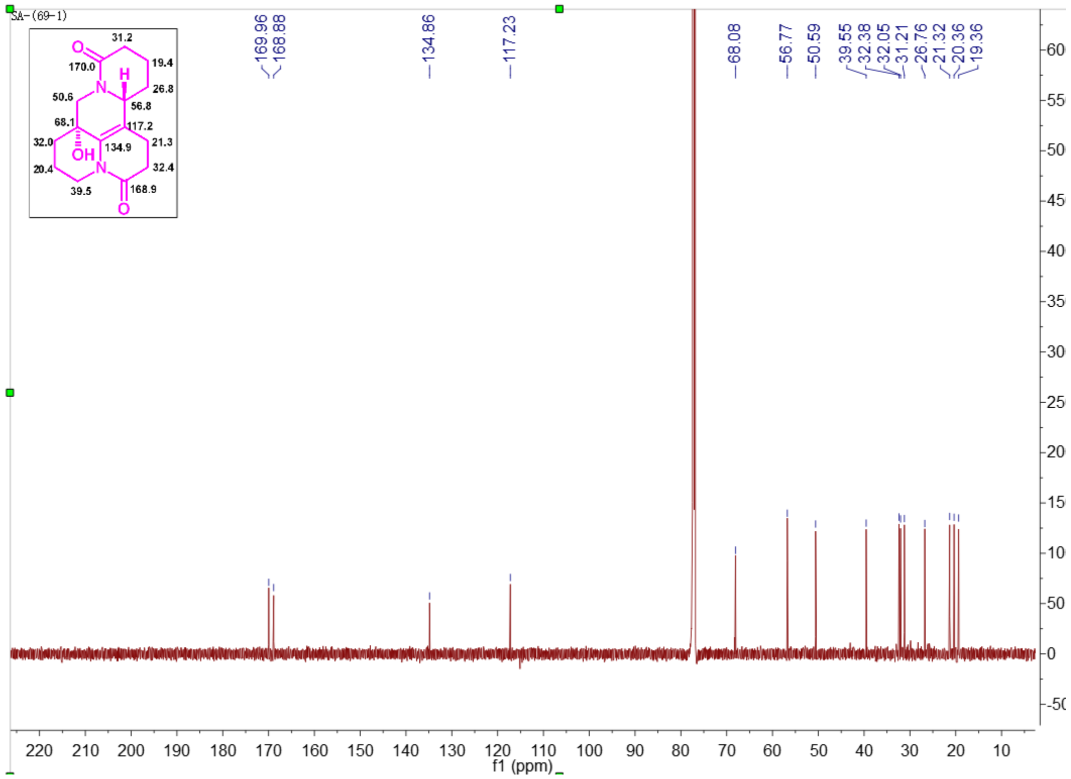

Figure S39. ^{13}**C NMR (150 MHz,** $CDCl_3$**) spectrum of 4**

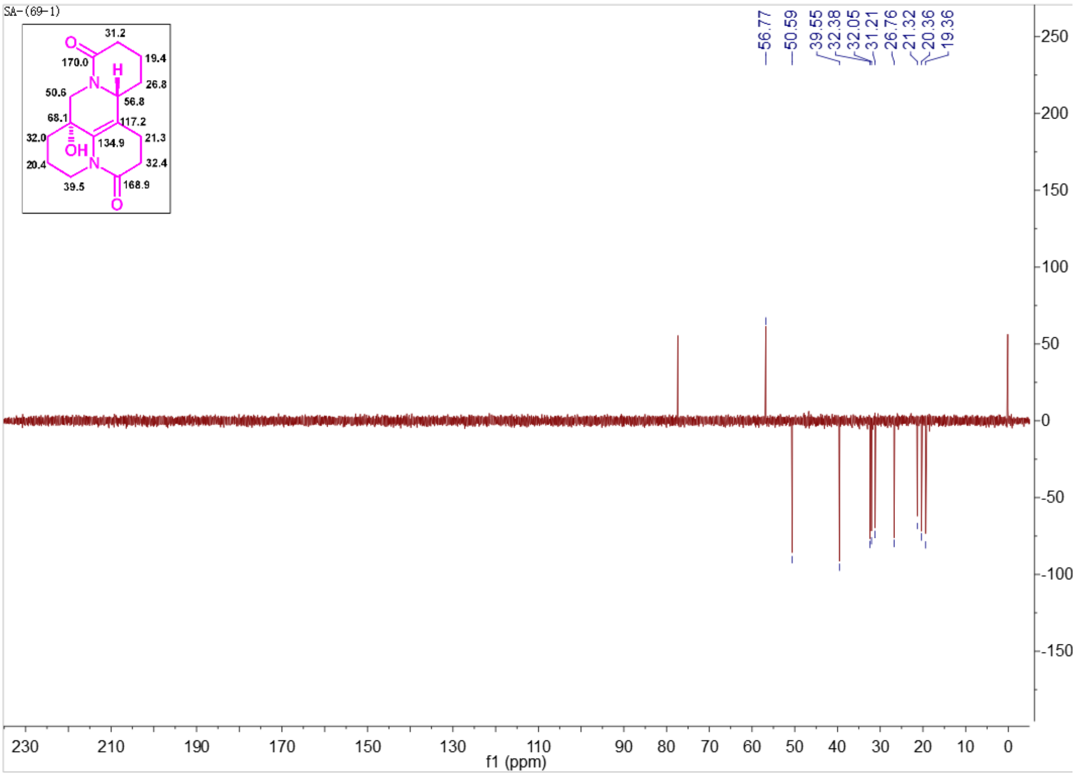

Figure S40. DEPT-135 spectrum of 4 inCDCl₃

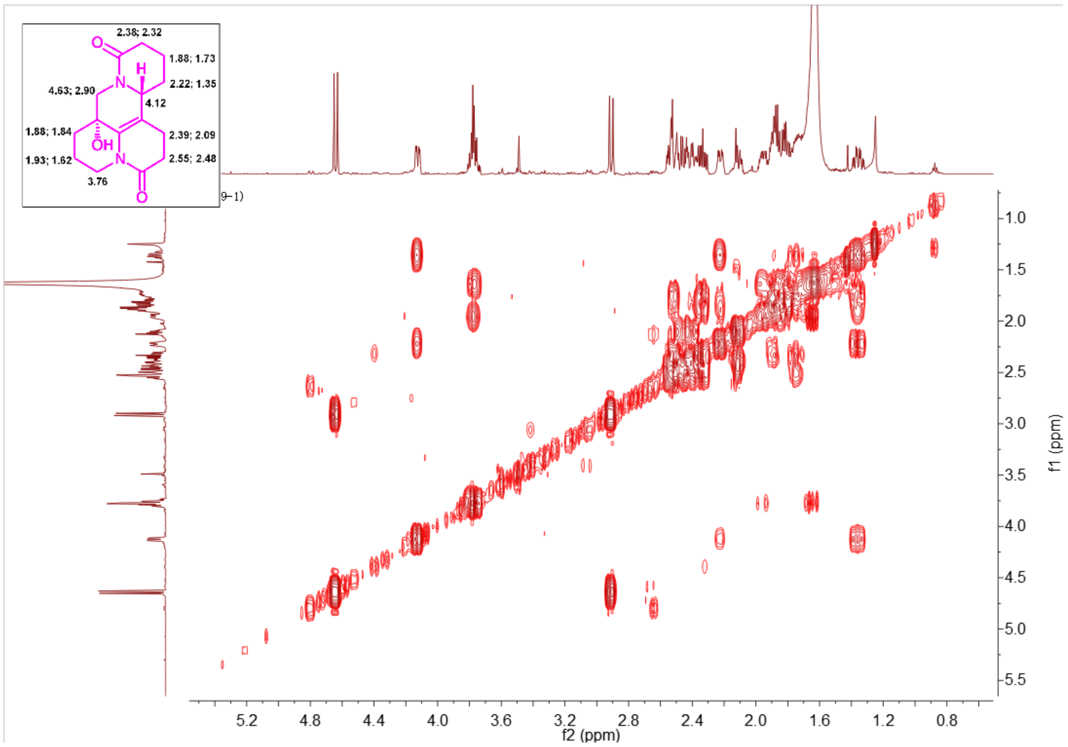

Figure S41. ^{1}H-^{1}H COSY spectrum of 4 inCDCl₃

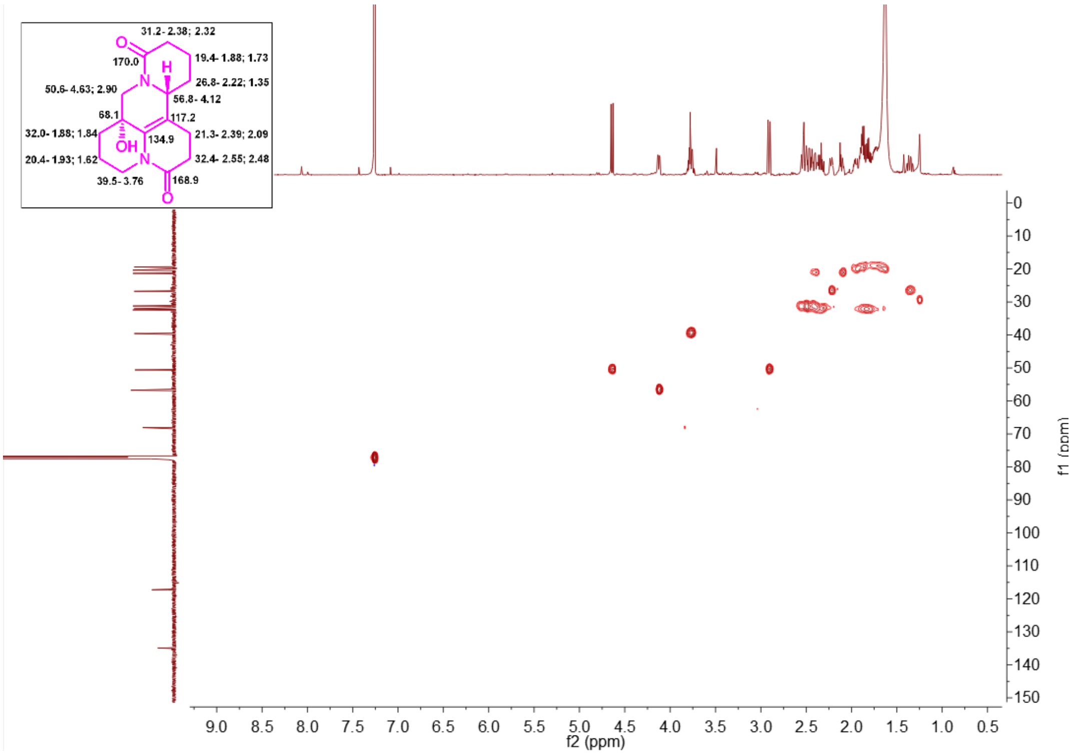

Figure S42. HSQC spectrum of 4 inCDCl3

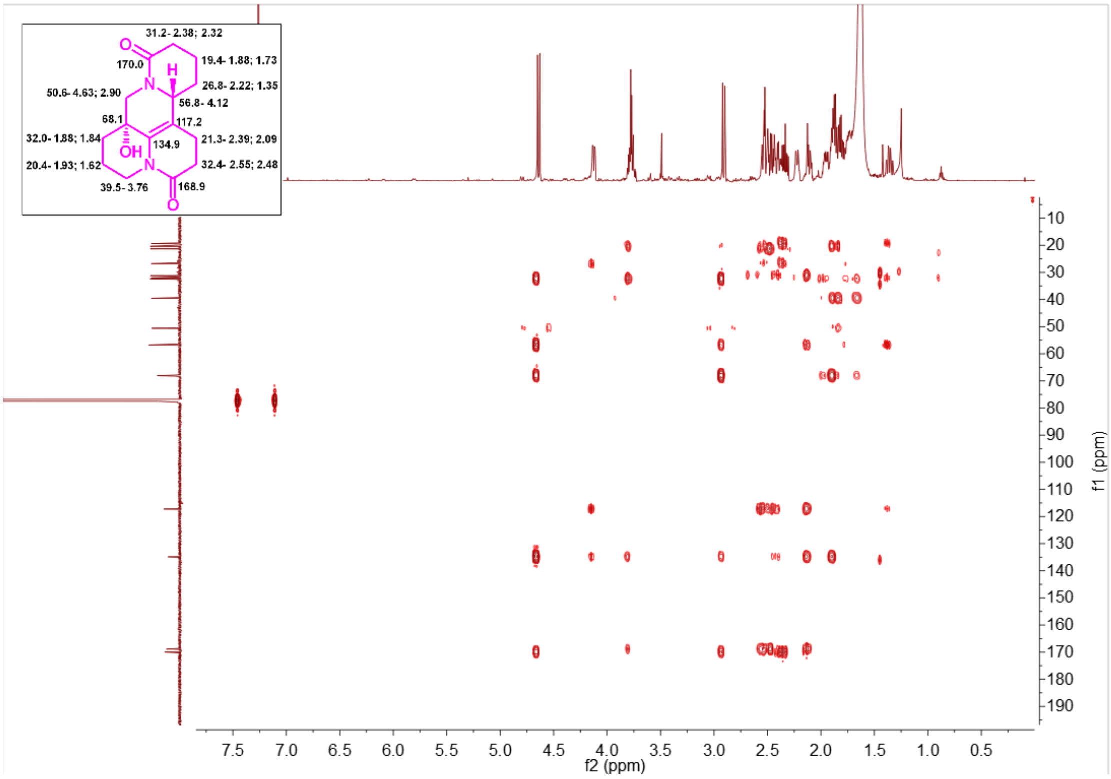

Figure S43. HMBC spectrum of 4 inCDCl3

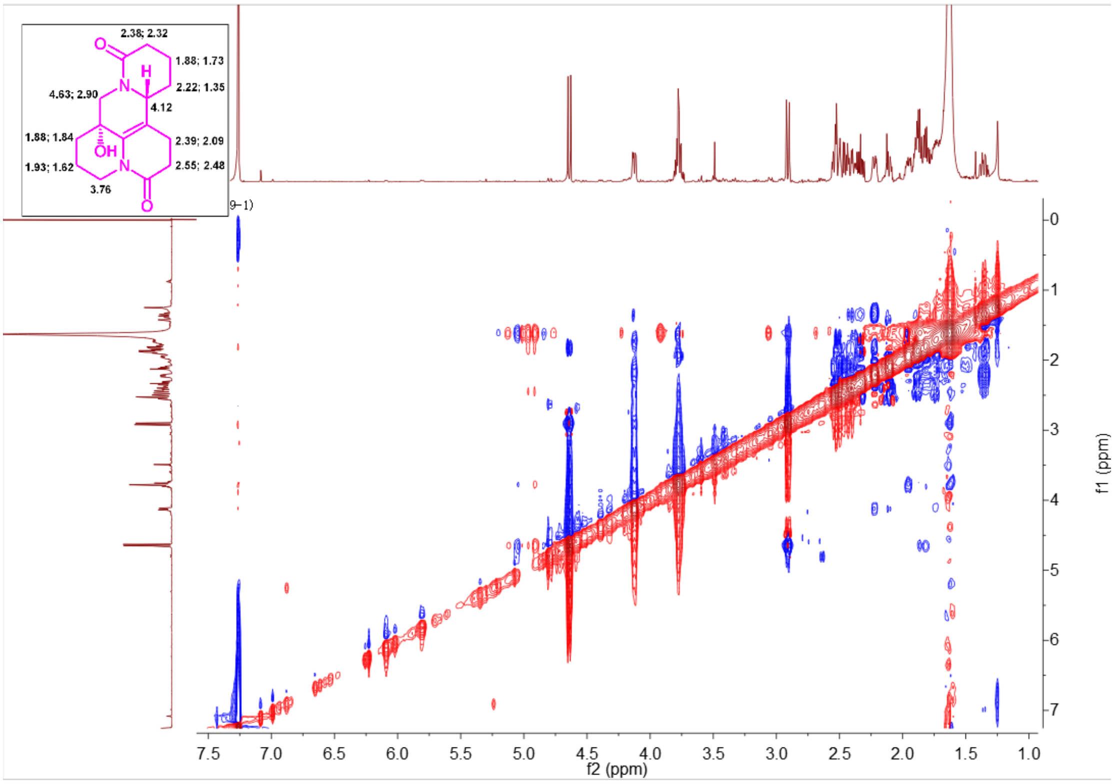

Figure S44. NOESY spectrum of 4 inCDCl$_3$

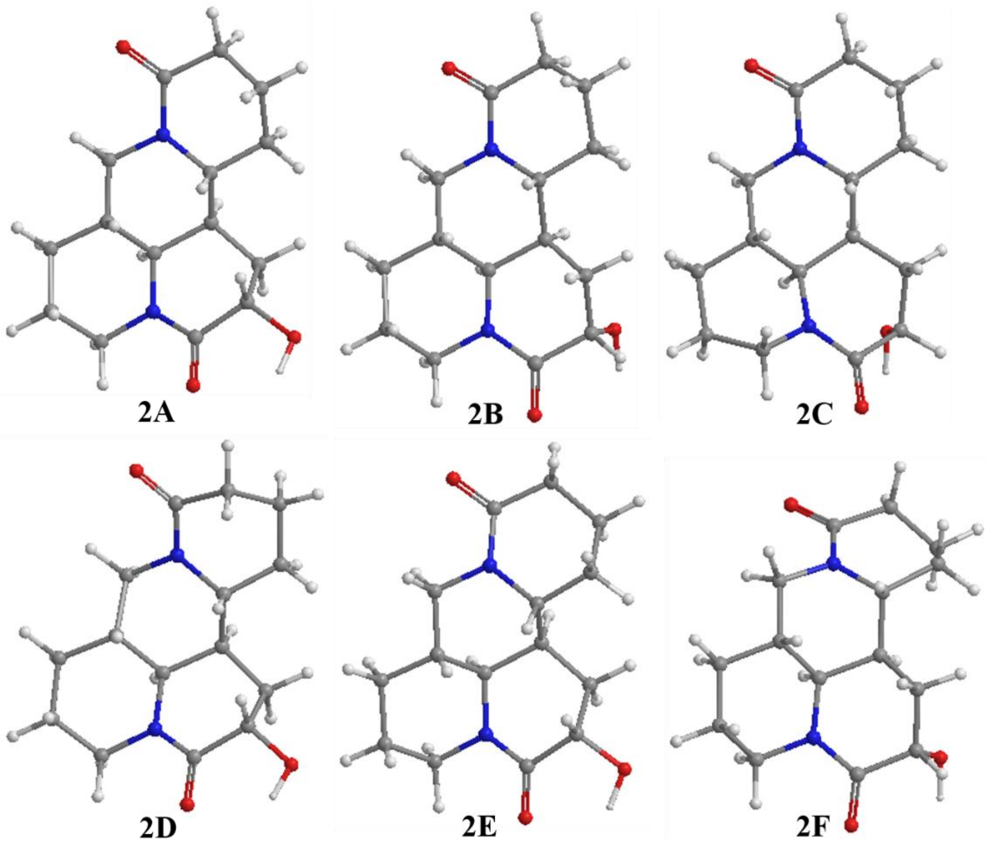

Figure S45. The predominant low-energy conformers of 2 at the B3LYP/6-31+G(d, p) level by Gaussian 09 program.

Table S1. Gibbs free energies and equilibrium populations of low-energy conformers of 2

Conformer name	B3LYP/6-31+G(d, p) Gibbs free energy (298.15 K)		
	**G (Hartree)**	**ΔG (Kcal/mol)**	**Boltzmann Distribution (%)**
**Conformer 2A**	-920.0902041	0.0000000	82.11
**Conformer 2B**	-920.0876336	0.0025705	13.63
**Conformer 2C**	-920.0869959	0.0032082	3.18
**Conformer 2D**	-920.0864604	0.0037437	1.02
**Conformer 2E**	-920.0854499	0.0047542	0.04

**Conformer 2F**	-920.0854291	0.0047750	0.02

Table S2. Standard orientations of Conformers 2A−2B.

**Conformer 2A**					
Center number	Atomic number	Atomic type	Coordinates (Angstroms)		
			X	Y	Z
1	6	0	0.954831	2.975851	-0.21149
2	6	0	2.429484	2.729083	-0.57102
3	6	0	3.046038	1.61599	0.288187
4	7	0	2.213879	0.40564	0.258362
5	6	0	0.825844	0.631579	0.70757
6	6	0	0.16718	1.657226	-0.23818
7	6	0	2.724504	-0.76521	-0.2005
8	6	0	1.777471	-1.96817	-0.23218
9	6	0	0.819651	-1.91253	0.952691
10	6	0	-0.0515	-0.64681	0.896195
11	6	0	-1.21678	-0.69658	-0.13262
12	7	0	-2.02115	0.542702	0.012964
13	6	0	-1.30316	1.827593	0.123122
14	6	0	-2.08756	-1.94805	0.027624
15	6	0	-3.39739	-1.8159	-0.74906
16	6	0	-4.20093	-0.64735	-0.18192
17	6	0	-3.39184	0.62847	0.002694
18	8	0	-3.95737	1.714316	0.14872
19	1	0	0.229869	1.257317	-1.26081
20	1	0	0.918183	1.113507	1.693564
21	1	0	-0.54369	-0.54463	1.871613
22	1	0	-0.8055	-0.71577	-1.15355
23	8	0	3.89534	-0.91078	-0.57075
24	8	0	2.534043	-3.15953	-0.21145
25	1	0	0.509305	3.697151	-0.90834
26	1	0	0.883884	3.421748	0.792467
27	1	0	3.015373	3.647699	-0.44173
28	1	0	2.508239	2.445203	-1.62913
29	1	0	4.041927	1.340122	-0.06028
30	1	0	3.127117	1.947896	1.334308
31	1	0	1.207937	-1.91413	-1.17703
32	1	0	1.415492	-1.90666	1.874294
33	1	0	0.21204	-2.82106	0.984817
34	1	0	-1.40986	2.224969	1.143563
35	1	0	-1.78836	2.551699	-0.53848

36	1	0	-2.30707	-2.11504	1.092845
37	1	0	-1.5216	-2.81632	-0.32486
38	1	0	-3.97584	-2.74541	-0.68782
39	1	0	-3.18114	-1.64751	-1.8136
40	1	0	-5.05906	-0.38513	-0.80908
41	1	0	-4.61057	-0.91039	0.803734
42	1	0	3.433175	-2.90701	-0.5047

Conformer 2B

Center number	Atomic number	Atomic type	Coordinates (Angstroms)		
			X	Y	Z
1	6	0	0.969772	2.899686	-0.33766
2	6	0	2.485179	2.705569	-0.44791
3	6	0	2.948702	1.604574	0.507987
4	7	0	2.235829	0.355561	0.212495
5	6	0	0.763827	0.441234	0.345096
6	6	0	0.196521	1.579779	-0.52387
7	6	0	2.96297	-0.79346	0.087038
8	6	0	2.223197	-2.0667	-0.32447
9	6	0	0.84017	-1.7912	-0.88177
10	6	0	0.032055	-0.90038	0.069427
11	6	0	-1.40528	-0.69324	-0.49009
12	7	0	-2.01893	0.541116	0.034301
13	6	0	-1.27537	1.799382	-0.15309
14	6	0	-2.27556	-1.92807	-0.2118
15	6	0	-3.75429	-1.65711	-0.48038
16	6	0	-4.22309	-0.54142	0.453896
17	6	0	-3.33568	0.694225	0.40225
18	8	0	-3.78371	1.805982	0.689063
19	1	0	0.281121	1.276828	-1.57829
20	1	0	0.570138	0.719477	1.394257
21	1	0	-0.05214	-1.43	1.024858
22	1	0	-1.32751	-0.57864	-1.58532
23	8	0	4.183971	-0.8491	0.265585
24	8	0	2.097571	-2.93236	0.809633
25	1	0	0.614734	3.641475	-1.06476
26	1	0	0.738688	3.305121	0.658798
27	1	0	3.006449	3.640919	-0.20804
28	1	0	2.766954	2.432441	-1.47389
29	1	0	4.015983	1.401285	0.418599
30	1	0	2.746991	1.896641	1.550945
31	1	0	2.852717	-2.54686	-1.08623
32	1	0	0.948908	-1.30483	-1.86128
33	1	0	0.331846	-2.74668	-1.05038
34	1	0	-1.3414	2.365163	0.782437

35	1	0	-1.77611	2.411997	-0.91508
36	1	0	-1.91082	-2.75828	-0.82588
37	1	0	-2.15165	-2.22974	0.837829
38	1	0	-4.34705	-2.56611	-0.32244
39	1	0	-3.90164	-1.36036	-1.52873
40	1	0	-5.23892	-0.20316	0.229003
41	1	0	-4.23467	-0.90058	1.493111
42	1	0	2.987681	-3.0563	1.180682

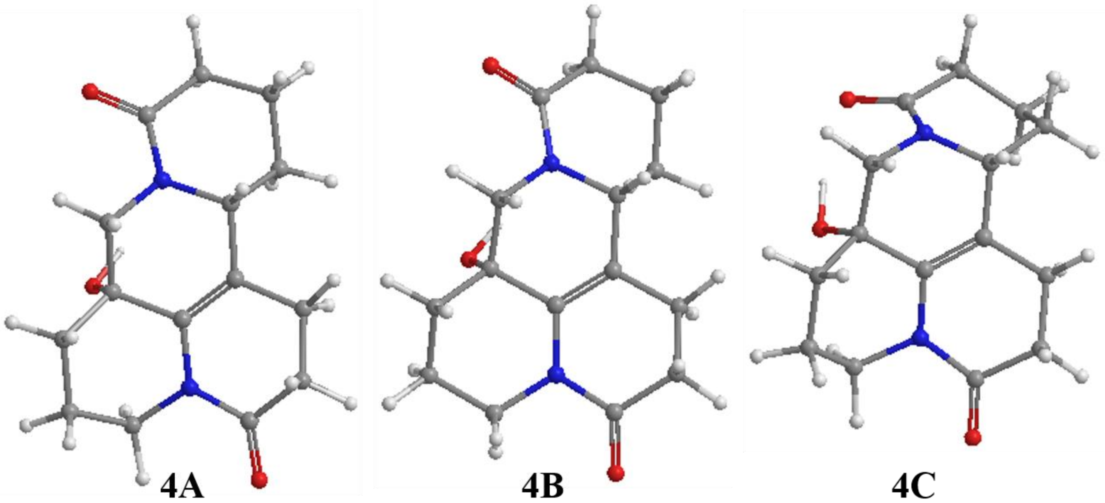

4A 4B 4C

Figure S46. The predominant low-energy conformer of 4 at the B3LYP/6-31+G(d, p) level by Gaussian 09 program.

Table S3. Gibbs free energies and equilibrium populations of low-energy conformer of 4

**Conformer name**	**B3LYP/6-31+G(d, p) Gibbs free energy (298.15 K)**		
	**G (Hartree)**	**ΔG (Kcal/mol)**	**Boltzmann Distribution (%)**
**Conformer 4A**	-918.8762019	0.0000000	97.91
**Conformer 4B**	-918.8720608	0.0041411	2.08
**Conformer 4C**	-918.8719265	0.0042754	0.01

Table S4. Standard orientations of Conformer 4.

Conformer 4A

Center number	Atomic number	Atomic type	Coordinates (Angstroms)		
			X	Y	Z
1	6	0	-1.43061	-2.29089	-0.80452
2	6	0	-2.78486	-2.29132	-0.05704
3	6	0	-2.96718	-1.04362	0.827993
4	7	0	-2.28862	0.129373	0.252937
5	6	0	-0.89988	0.015991	-0.00736
6	6	0	-0.37897	-1.41139	-0.11987
7	6	0	-2.99512	1.292959	0.012541
8	6	0	-2.1913	2.407271	-0.64193
9	6	0	-0.74001	2.470388	-0.15172
10	6	0	-0.11218	1.094778	-0.1811
11	6	0	1.378924	0.993489	-0.43872
12	7	0	1.840663	-0.40393	-0.33675
13	6	0	0.931149	-1.39109	-0.91574
14	6	0	2.194792	1.905638	0.491834
15	6	0	3.689584	1.722276	0.22997
16	6	0	4.089122	0.270879	0.50281
17	6	0	3.144279	-0.78154	-0.06683
18	8	0	3.525912	-1.94075	-0.21103
19	8	0	-0.15585	-1.98555	1.182125
20	1	0	1.565251	1.33969	-1.47578
21	8	0	-4.19065	1.405944	0.26529
22	1	0	-1.03325	-3.30825	-0.89122
23	1	0	-1.56768	-1.90989	-1.82383
24	1	0	-3.59877	-2.3533	-0.78851
25	1	0	-2.86354	-3.17455	0.585196
26	1	0	-4.01751	-0.77517	0.938611
27	1	0	-2.54539	-1.22623	1.821051
28	1	0	-2.20528	2.219647	-1.72602
29	1	0	-2.72667	3.343904	-0.46785
30	1	0	-0.17525	3.168799	-0.78245
31	1	0	-0.70457	2.885193	0.868375
32	1	0	1.416063	-2.36634	-0.86865
33	1	0	0.731247	-1.14212	-1.96868
34	1	0	1.957011	1.663961	1.537404
35	1	0	1.897932	2.94623	0.32417
36	1	0	3.91169	1.985443	-0.8141
37	1	0	4.280372	2.400837	0.856818
38	1	0	5.086579	0.035874	0.119397

39	1	0	4.127511	0.08848	1.58657
40	1	0	0.598176	-1.52036	1.583199
